# Lomitapide, a cholesterol-lowering drug, is an anticancer agent that induces autophagic cell death via inhibiting mTOR

**DOI:** 10.1038/s41419-022-05039-6

**Published:** 2022-07-12

**Authors:** Boah Lee, Seung Ju Park, Seulgi Lee, Jinwook Lee, Eunbeol Lee, Eun-Seon Yoo, Won-Suk Chung, Jong-Woo Sohn, Byung-Chul Oh, Seyun Kim

**Affiliations:** 1 Department of Bio and Brain Engineering, Korea Advanced Institute of Science and Technology (KAIST), Daejeon, 34141 Korea; 2 Department of Biological Sciences, KAIST, Daejeon, 34141 Korea; 3 Department of Physiology, Lee Gil Ya Cancer and Diabetes Institute, Gachon University, College of Medicine, Incheon, 21999 Korea; 4 KAIST Institute for the BioCentury, KAIST, Daejeon, 34141 Korea; 5 KAIST Stem Cell Center, KAIST, Daejeon, 34141 Korea; 6 Present Address: ERSTEQ co., Ltd, Daejeon, 34013 Korea

**Keywords:** Drug development, Drug development

## Abstract

Autophagy is a biological process that maintains cellular homeostasis and regulates the internal cellular environment. Hyperactivating autophagy to trigger cell death has been a suggested therapeutic strategy for cancer treatment. Mechanistic target of rapamycin (mTOR) is a crucial protein kinase that regulates autophagy; therefore, using a structure-based virtual screen analysis, we identified lomitapide, a cholesterol-lowering drug, as a potential mTOR complex 1 (mTORC1) inhibitor. Our results showed that lomitapide directly inhibits mTORC1 in vitro and induces autophagy-dependent cancer cell death by decreasing mTOR signaling, thereby inhibiting the downstream events associated with increased LC3 conversion in various cancer cells (e.g., HCT116 colorectal cancer cells) and tumor xenografts. Lomitapide also significantly suppresses the growth and viability along with elevated autophagy in patient-derived colorectal cancer organoids. Furthermore, a combination of lomitapide and immune checkpoint blocking antibodies synergistically inhibits tumor growth in murine MC38 or B16-F10 preclinical syngeneic tumor models. These results elucidate the direct, tumor-relevant immune-potentiating benefits of mTORC1 inhibition by lomitapide, which complement the current immune checkpoint blockade. This study highlights the potential repurposing of lomitapide as a new therapeutic option for cancer treatment.

## Introduction

Macroautophagy (hereafter referred to as autophagy) is a highly dynamic catabolic process involving the degradation of damaged organelles, misfolded proteins, and long-lived macromolecules in lysosomes [1]. Under basal conditions, this process degrades long-lived proteins; however, when cells are under stress, such as during starvation or hypoxia, autophagy is drastically elevated to enhance cell survival, thereby acting as a protective mechanism [2]. Autophagy is an orchestrated process involving several steps, initiated by the formation and elongation of the phagophore, which subsequently expands by acquiring lipids, and ultimately transforms into a completely sealed double-membrane structure called the autophagosome [3]. The autophagosome then fuses with the lysosome to form the autolysosome, where the sequestered cargo is degraded and recycled. This recycling process enables the cells to cope with various stress conditions and maintain cellular homeostasis. Autophagy is also involved in the progression of numerous disorders, such as cancer, auto-immune diseases, infections, and neurodegeneration [4–6].

In the context of cancer, regulating autophagy can be a double-edged sword [7–9]. On one hand, autophagy can become a crucial survival mechanism for tumor cells under various stresses. Activation of autophagy has been reported to have a protective effect on cancer cells undergoing anticancer treatments facing various stressful conditions, thereby leading to poor treatment outcomes and the development of treatment resistance [10, 11]. On the other hand, emerging evidence has indicated that excess autophagy can lead to autophagic cell death [12–14], also known as type II programmed cell death [15]. Preclinical studies have shown that genetic or pharmacological hyperactivation of autophagy can promote tumor regression, highlighting the potential of targeted autophagy as an effective therapeutic strategy for cancer [9, 16, 17]. Autophagic cell death can be triggered in different cancer cell types by various compounds including BH3 mimetics such as obatoclax and gossypol, histone deacetylase inhibitors, as well as natural plant products such as resveratrol and betulinic acid [18, 19].

The mTOR complex is the most important regulator of autophagy [20]. mTOR is a serine/threonine kinase that crucially functions as a cellular signaling network node, wherein extracellular and intracellular conditions are integrated by including growth factors, cellular stressors, and nutrients such as amino acids [21]. Therefore, mTOR signaling mediates a plethora of major biological events involved in growth and metabolism [22, 23]. Throughout extensive protein–protein interactions, mTOR exists as two multi-subunit complexes: mTOR complex 1 (mTORC1) and 2 (mTORC2). Activation of mTORC1 has been reported to promote cell growth by phosphorylating S6 kinase 1 (S6K1) and 4E-BP1 [24]. Stimulation of mTORC2 has been reported to lead to cell survival and actin cytoskeletal changes by phosphorylating Akt, protein kinase C, and serum/glucocorticoid regulated kinase 1 [20, 21]. Under nutrient-replete conditions, mTOR has been reported to block the initiation of autophagy by phosphorylating ULK1 [25]. Starvation or the pharmacologic inhibition of mTOR can cause its dissociation from the complex of ATG13 with ULK1 and ULK2, thereby triggering autophagosome formation and autophagy [26]. The dysregulation of the mTOR signaling pathway has also been linked with cancer, inflammation, diabetes, and neurological diseases [27, 28]. In fact, 70% of all known cancers have been shown to be associated with aberrant hyperactivation of mTOR, which promotes cellular proliferation and delays the apoptosis of tumor cells [29, 30]. Therefore, regulating the mTOR signaling pathway can result in cancer cell death with elevated autophagy, thereby highlighting its potential in the development of new cancer treatments [31, 32].

Identifying therapeutic approaches to treat cancer is laborious, expensive, and often inefficient. Drug repurposing or repositioning in oncology refers to the application of drugs, which are already approved for other medical applications, in treating cancer. Compared to de novo drug discovery, the development risks, costs, and chances of safety-related failures are reduced with the use of repurposed drugs because their thoroughly researched pharmacokinetic and pharmacodynamic profiles are largely accessible [33]. Moreover, in order to enhance therapeutic benefits, repurposed drugs are often combined with frequent administrations of low-dose chemotherapy. Recent advancements in structure-based molecular docking and computational analyses have led to the development of in silico drug discovery approaches. Therefore, therapeutic discovery through a drug repurposing strategy aided by these technological advancements can potentially accelerate studies into clinical trials more rapidly compared to that using newly developed drugs.

In this study, we employed a structure-based virtual screening approach to identify an mTOR inhibitor candidate. Using in vivo cellular and biochemical experiments as well as transcriptome sequencing analyses, we identified lomitapide, an inhibitor of hepatic microsomal triglyceride transfer protein (MTTP) is an mTOR inhibitor. Lomitapide is an effective and well-tolerated cholesterol-lowering drug approved for the treatment of homozygous familial hypercholesterolemia (HoFH), a rare genetic disorder of low-density lipoprotein cholesterol (LDL-C) metabolism resulting in extremely elevated serum levels of LDL-C and premature atherosclerotic cardiovascular disease [34, 35]. Mechanistically, lomitapide directly inhibits the kinase activity of mTOR and induces autophagy, thereby suppressing growth while increasing cancer cell death. Our results indicate that the U.S. FDA-approved drug, lomitapide, can be potentially repurposed for the treatment of cancer.

## Materials and methods

### Ethics statement

All mice were housed in a pathogen-free animal facility at KAIST Laboratory Animal Resource Center. The animals were maintained in a temperature/humidity-controlled room on a 12 h light/12 h dark cycle and fed a standard chow diet. All experiments involving animals were conducted according to the ethical policies and procedures approved by the Committee for Animal Care at KAIST.

### Molecular modeling

Docking simulations using the Libdock algorithm [36] in Discovery Studio 3.1 (Accelrys Inc., USA) were performed with compounds. The X-ray crystal structure complex of ATP-bound human mTORC1 (PDB ID: 4JSV) and cryo-EM structure of human mTORC2 (PDB ID: 5ZCS) were obtained from the protein data bank. The proposed binding site for mTORC1 was centered on the ligand and a site sphere was created at coordinates −19.17, −31.85, and −58.25 with a 14.58 Å diameter and for mTORC2, a sphere was generated at coordinates 196.59, 165.31, 217.46 with a 16.34 Å diameter including ATP-binding residues according to the structure of mTORC1. The protocols included 100 hotspots with a docking tolerance of 0.25. The FAST confirmation method was also used with CHARMM.

### Fluorescence-based thermal shift assay

The thermal shift assays were performed using the 7500 Real-Time PCR System (Applied Biosystems, USA) melting curve program with a temperature increment of 1.0 °C and a temperature range of 25–95 °C. All reactions were incubated in a 20 μl final volume and assayed in 96-well plates using 1:1,000 dilution of 5000 × SYPRO Orange stock solution (Sigma-Aldrich, USA) and indicated concentrations (1.0 μM) of recombinant mTOR kinase domain diluted in buffer containing 10 mM HEPES·HCl pH 7.5. Lomitapide was added to the reaction to assess ligand-dependent thermal destabilization of mTOR kinase domain protein. The ligands (dissolved in DMSO) were incubated with mTOR kinase domain protein at 4 °C for 25 min before acquiring the melting curves [37]. The Tm is identified by plotting the first derivative of the fluorescence emission as a function of temperature (−dF/dT) using GraphPad PRISM7 software.

### In vitro mTOR kinase assay

For in vitro mTOR kinase assay, cells were rinsed once with ice-cold PBS and lysed in ice-cold CHAPS buffer. Cell lysates were incubated at 4 °C for 10 min and the supernatant was collected by centrifuging lysates at 13,000 rpm for 10 min. Two micrograms of mTOR antibody (#2972, Cell Signaling Technology, USA) were added to the 2 mg of cell lysates and incubated with rotation for 2 h at 4 °C. About 20 ml of agarose beads (Pierce, USA) were added and the incubation continued for an additional 1 h. mTOR immunoprecipitates were washed twice with the same lysis buffer and twice with kinase wash buffer (25 mM HEPES at pH 7.4, 20 mM potassium chloride, and 1 mM magnesium chloride). Kinase assays were performed for 15 min at 37 °C in a final volume of 15 ml of mTORC1 kinase buffer (25 mM HEPES at pH 7.4, 50 mM KCl, 10 mM MgCl_2_, 500 μM ATP) and 150 ng of S6K1 as a substrate. Reactions were stopped by the addition of 10 ml of sample buffer and boiling for 5 min and analyzed by SDS-PAGE and immunoblotting. In vitro mTORC2 kinase assay was performed by using mTORC2 kinase buffer (25 mM HEPES at pH 7.5, 100 mM potassium acetate, 1 mM MgCl_2_, 500 μM ATP) with 100 ng of Akt1 as a substrate.

### Cell lines and culture conditions

HCT116 cells (human colon cancer cells, p53 wildtype), HT29 cells (human colon cancer cells, p53 mutant), SW480 cells (human colon cancer cells, p53 mutant), MDA-MB-231 cells (human breast cancer cells), MDA-MB-468 cells (human breast cancer cells), A375 (human skin cancer), and A2058 (human skin cancer) were purchased from ATCC (American Type Culture Collection, Virginia, USA). NCM460 cells (normal human colon mucosa cells) were obtained by a cell licensing agreement with INCELL Corporation (USA). HS756T (human stomach cancer), SNU1 (human stomach cancer), and SNU216 (human stomach cancer) cells were purchased from Korea Cell Line Bank (Korea). HCT116 cells were cultured in McCoy’s 5a medium (Sigma-Aldrich) supplemented with 2 mM glutamine, 1% penicillin-streptomycin, and 10% fetal bovine serum (FBS) at 37 °C and 5% CO_2_. HT29 and SW480 cells were cultured in RPMI medium (Sigma-Aldrich) supplemented with 2 mM glutamine, 1% penicillin-streptomycin, and 10% FBS at 37 °C and 5% CO_2_. MDA-MB-231, MDA-MB-468, A375, A2058, HS-746T, SNU1, and SNU216 cells were supplemented with 2 mM glutamine, 1% penicillin-streptomycin and 10% FBS in DMEM medium (Sigma-Aldrich) at 37 °C and 5% CO_2_.

### Cancer cell viability screen

High-throughput cancer cell viability assays were performed by Reaction Biology Corp. (USA). About 120 major cancer cell lines derived from skin, breast, brain, ovary, liver, stomach, kidney, bone, pancreas, intestine, lung, and blood cancer were inoculated in a 96-well plate at a density of 10^4^ cells/well, and cultured at 37 °C for 24 h, followed by various concentrations (0, 1, 2, 5, 10 µM) of lomitapide (Sigma-Aldrich). The plate was incubated at 5% CO_2_ at 37 °C for 24 h after treatment with lomitapide. Thereafter, cells were added to each well of 100 µl of the assay reagent (CellTiter-Glo^®^ Reagent), and luminescence was measured using a VICTOR X Multilabel Reader (PerkinElmer, USA).

### Colony-forming assay

In order to test the action of lomitapide in the control of cancer cell proliferation in the HCT116, HT29, and SW480 cell lines, the rate of cancer cell colony proliferation was examined by adding lomitapide to the wells in which cells were cultured. HCT116, HT29, and SW480 cells were inoculated in a 12-well plate at a density of 10^5^ cells/well and incubated at 37 °C for 24 h, and then the cells were treated with 0, 5 μM concentration of lomitapide. After lomitapide treatment, the plate was incubated at 5% CO_2_ at 37 °C for 48 h. To measure colony formation of HT29^FLAG-elF4E-GFP^ stable cells, cells were seeded in a 12-well plate at a density of 1.25 × 10^4^ cells/well and incubated at 37 °C overnight, and then the cells were treated with a 2 μM concentration of lomitapide, rapamycin (Merck KGaA, Germany), and PP242 (Selleckchem, USA) for 96 h. Thereafter, 500 μl of crystal violet was added to each well, and cells were stained at room temperature for 10 min to analyze cell proliferation.

### Immunoblotting

Levels of signaling protein expression and activity were measured with immunoblotting in various cancer cell lines. Lomitapide-treated cells at various concentrations (0, 5, 10 μM) were lysed with RIPA buffer (50 mM Tris-Cl pH 7.5, 150 mM NaCl, 1 mM EDTA, 50 mM sodium fluoride, 10 mM sodium pyrophosphate, 10 mM glycerophosphate, 1% NP-40, 0.25% sodium deoxycholate, 0.1% SDS) containing protease-inhibitor cocktail. Whole-cell lysate was incubated on ice for 30 min, then centrifuged at 4 °C, 13,300 × *g* for 15 min and the supernatant was collected. As a control, cells treated with 1 µM of Torrin1, known as an mTOR inhibitory compound, were used. For immunoblot analysis, the supernatant obtained above was loaded on a 10% SDS-PAGE gel to separate, and the separated protein was blotted onto a nitrocellulose membrane. Anti-p-Akt (#9271, 1:1000), anti-p-mTOR (#5536, 1:1000), anti-p-S6K (#9205, 1:500), anti-p-S6 (#5364, 1:1000), anti-p-Erk (#9101, 1:1000), anti-Akt (#9272, 1:1000), anti-S6K (#9202, 1:1000), anti-S6 (#2217, 1:1000), anti–Erk (#9102, 1:1000), anti-p-4E-BP1 (#9459, 1:1000), anti-4E-BP1 (#9452, 1:1000), anti-p-ULK1 (#6888, 1:1000), anti-ULK1 (#8054, 1:1000), anti-LC3 (#2775, 1:1000), anti-p-MEK (#9154, 1:1000), anti-MEK (#4694, 1:1000), anti-p-PTEN (#9554, 1:1000), anti-PTEN (#9559, 1:1000), anti-eIF4E (#9742, 1:1000), anti-β-actin (#4970, 1:1000), anti-AMPK (#5831, 1:1000), anti-TSC1 (#6935, 1:1000), anti-TSC2 (#3612, 1:1000), anti-PLD1 (#3832, 1:1000), and anti-PLD2 (#13904, 1:1000) (Cell Signaling Technology, USA), anti-alpha-tublin antibody (Sigma-Aldrich, T5168), and anti-GAPDH (sc-32233), anti-HSP90 (sc-13119) (Santa Cruz Biotechnology, USA) were used. Antibodies were added and incubated at 4 °C for overnight. The blot was then washed with a mixture of tris-buffered saline (TBS) and Tween-20 (TBST) and horseradish peroxidase-conjugated secondary antibody (Cell Signaling Technology) at 37 °C. After incubation and washing for 1 h, enhanced chemiluminescence (Bio-Rad, USA) was detected.

### RNA-sequencing analysis

Total RNA was isolated from tissue using Maxwell (Promega, USA) based method. One microgram of total RNA was processed for preparing the mRNA sequencing library using MGIEasy RNA Directional Library Prep Kit (MGI, China) according to the manufacturer’s instruction. The first step involves purifying the poly-A-containing mRNA molecules using poly-T oligo-attached magnetic beads. Following purification, the mRNA is fragmented into small pieces using divalent cations under elevated temperatures. The cleaved RNA fragments are copied into first-strand cDNA using reverse transcriptase and random primers. Strand specificity is achieved in the RT directional buffer, followed by second-strand cDNA synthesis. These cDNA fragments then have the addition of a single ‘A’ base and subsequent ligation of the adapter. The products are then purified and enriched with PCR to create the final cDNA library. The double-stranded library is quantified using QauntiFluor ONE dsDNA System (Promega). The library is circularized at 37 °C for 30 min and then digested at 37 °C for 30 min, followed by a cleanup of the circularization product. To make DNA nanoball (DNB), the library is incubated at 30 °C for 25 min using the DNB enzyme. Finally, Library was quantified by QauntiFluor ssDNA System (Promega). Sequencing of the prepared DNB was conducted on the MGIseq system (MGI) with 150 bp paired-end reads. The limma, edgeR, msigdbr, clusterProfiler packages in R, an open-source programming environment, was used to perform differentially expressed genes, gene set enrichment, and pathway enrichment analysis. ENTREZID, MsigDB, GO terms, and KEGG pathways were mapped and were used to perform enrichment tests based on the hypergeometric distribution. To prevent a high false discovery rate (FDR) in multiple testing, *q* values were also estimated for FDR control. Sequence data were submitted to the NCBI Sequence Read Archive under BioProject ID PRJNA837533.

### Autophagy assays

To confirm the association of lomitapide’s ability to induce autophagy, 3-methylamine (3-MA) and bafilomycin (Sigma-Aldrich) were used. LC3 level was determined under lomitapide treatment in the absence or presence of 1 mM 3-MA or bafilomycin. Cell viability was measured using CellTiter-Glo^®^ Reagent.

### Immunofluorescence

HT29 and HCT116 cells were fixed with 4% paraformaldehyde, permeabilized with 0.1% TrintonX-100 in DPBS, and blocked with 3% goat serum. Then cells were stained with anti-GFP, anti-LC3B (Cell Signaling Technology), and anti-LAMP2 antibody (Santa Cruz Biotechnology), and counterstained with DAPI (Thermo Fisher Scientific, USA). Image taking and processing were carried out with laser scanning confocal microscopy (Carl Zeiss AG, Germany). Visualization and picture in the same panel were taken under the same excitation conditions. Fresh frozen tumor tissues were sectioned at 10 μm with a cryostat. Anti-CD8, anti-PD-L1, anti-CD8, and DAPI were used for detection.

### Caspase 3/7 assays

Caspase activity was measured from HCT116 and HT29 cells treated with lomitapide for 24 h. Etoposide was used as control for the induction of apoptosis. Z-VAD was used as a control for the inhibition of apoptosis.

### Viability assay of organoids derived from colorectal cancer patients

The anticancer effect of lomitapide on CRC organoids were analyzed by Organoid Sciences (Korea). Organoids derived from colorectal cancer patients (CRC-01 from a 46-year-old male, CRC-02 from a 74-year-old female) were cultured for 5–7 days in 48-well plates. Cytation5 (Biotek, USA), a high-content imaging-based screening device, was used to analyze organoids. After removing the culture medium from the organoids so that they would not separate from the plate, the organoids were transferred to new tubes by pipetting them with 1,000 μl of DPBS. The tubes with organoids were centrifuged at 1350 rpm for 5 min, and the supernatant was removed. Organoids were stained with Hoechst33342 (#H-1339; Thermo Fisher Scientific) for 30 min at 37 °C in a 5% CO_2_ incubator. After staining, the tube with organoids were centrifuged at 1350 rpm for 5 min, and the supernatant was removed. Next, 100 μl of organoid culture solution was added to the organoids, mixed with the organoids via pipetting, and centrifuged at 1350 rpm for 5 min, the supernatant was removed. Cell pellets were resuspended in a 1:1 mixture of growth medium and Matrigel and then seeded in 96-well black plates at a density of 150–200 cells/well. The Matrigel was polymerized for 10 min at 37 °C, and the culture medium with PI and drugs was added to the wells. Cytation5 (Biotek) was used to identify the number, morphology, and area of organoids via a DAPI signal. Then, without changing the culture solution, changes in the organoid area were observed every 24 for 72 h. Based on the raw data from the Cytation5 device, the efficacy of the drug was calculated using the formula below. The overall efficacy of a drug at a specific concentration is defined as the percentage of organoid growth inhibition and organoid death. The area of organoids stained with Hoechst33342 (μm^2^) was observed with Cytation5, and the areas of all organoids in each well were added. The difference in organoid areas was calculated by subtracting the initial area of the organoids (at 0 h post-treatment) from the final area of the organoids (at 72 h post-treatment).

### In vivo xenograft assay

In order to confirm the effect of lomitapide anticancer in a mouse xenograft model, changes in tumor size were examined after treatment with lomitapide in mice transplanted with tumors. HCT116 (2 × 10^6^) and HT29 (5 × 10^6^) cells were implanted subcutaneously into 6–8 weeks old male or 5–6 weeks old female BALB/c nude mice respectively. After the average tumor volume reached 50 mm^3^, mice were randomly assigned to two different groups (six animals/group). Mice’s body weight and tumor diameter were measured once every other day. Tumor volume was evaluated according to the general formula 0.5 × (width)^2^ × (length) using a caliper, and Student’s *t*-test was used to determine *P* values. For treatment with lomitapide, 10, 20, 25, and 50 mg/kg of lomitapide was injected intraperitoneal into mice as indicated. The experiment was conducted in the same way as above for 10 days at 2 days intervals, also the intratumoral injection method was applied for 10 days at five injection intervals. The investigator was blinded to the group allocation of the animals during the experiment. No statistical method was used to predetermine the sample size for the animal experiment, which was based on previous experimental observations. The sample size of each experiment is shown in the legend. No data were excluded from the analysis.

### Immunohistochemistry

Tissues and tumors were embedded in paraffin, and 5 µm sections were prepared and stained with Hematoxylin and eosin Y solution (H&E) by the KPNT (Korea Pathology Technical Center, Korea). For immunohistochemical staining of Ki67, we used paraffin-embedded sections (5 μm) of mouse tumor tissue. We performed heat-mediated antigen retrieval in citrate buffer (pH 6.0). Deparaffinized tissue sections were incubated with the primary antibody of Ki67 (ab15580 Abcam). Staining was visualized by using mouse-specific HRP/DAB detection IHC kit (ab64259 Abcam) and sections were counterstained with Mayer’s hematoxylin. Sections were photomicrographed with a digital camera mounted on a light microscope (Olympus BX51, Japan), digitized, and analyzed. Analysis was performed on 10 fields of a section at 40× magnification.

### Mouse tumorigenesis and treatment

For the syngeneic tumor mouse model, experiments were conducted using two types of MC38 colorectal cancer and B16-F10 cutaneous melanoma cell lines in 6-week-old, wild-type female or male C57B6/N mice (Orient Bio Inc., Korea) respectively. Before tumor cell injection, mice were randomized into four different groups (ten in each group). MC38 colorectal cancer and B16-F10 cutaneous melanoma cells were injected subcutaneously at 2 × 10^5^. Lomitapide used in both models was formulated with 45% saline, 40% PEG300, 5% Tween-80, and 10% DMSO. For MC38 colorectal cancer, lomitapide was administered intraperitoneally at a dose of 20 mg/kg from 10 days after tumor injection for five doses, and 10 mg/kg anti-PD-1 mAb (clone RMP1–14, BioXCell, USA) or a rat IgG2a isotype control (clone 2A3, BE0089; BioXCell) were administered on days 1, 4, 7, and 10 in PBS. In the case of B16-F10 cutaneous melanoma, lomitapide was administered at a dose of 20 mg/kg five times intraperitoneally every other day from 10 days after tumor injection, and 7.5 mg/kg anti-PD-1 mAb (clone RMP1–14, BioXCell) or rat IgG2a isotype control (clone 2A3, BioXCell) were administered on days 1, 4, 7, and 10 in PBS. Mice were immediately euthanized when signs of distress were observed, 20% weight loss of normal body weight or tumor volume exceeded 1000 mm^3^. The investigator was blinded to the group allocation of the animals during the experiment. No statistical method was used to predetermine the sample size for the animal experiment, which was based on previous experimental observations. The sample size of each experiment is shown in the legend. No data were excluded from the analysis.

### Generation of stable cell lines

The plasmid pCDH-MCS-T2A-copGFP-MSCV (CD525A-1; System Biosciences, USA) was used as a backbone for expression of eIF4E (#33252; Addgene, USA). Lentiviruses were generated in HEK 293 T cells by transfecting the lentiviral plasmids together with packaging vectors (pRSV-Rev, pMDLg/pRRE) and envelop expressing plasmid (pMD2.G) as the manufacturer’s protocol. Lentiviruses were added to HT29 cells for 48 h and the transduced cells were subjected to be analyzed for drug treatment, cell viability assay, and colony-forming assay.

### Phospholipase D (PLD) enzyme activity assays

After HT29 cells were treated with DMSO, 10 μM lomitapide, 10 μM 5-Fluoro-2-indolyl des-chlorohalopemide hydrochloride hydrate (FIPI) for 4 h. Cells were lysed with lysis buffer (50 mM Tris-Cl pH 7.5, 150 mM NaCl, 1 mM EDTA, 50 mM sodium fluoride, 10 mM sodium pyrophosphate, 1% TritonX 100) containing protease-inhibitor cocktail. About 2 mg of whole-cell lysate was added to 2 μg of anti-PLD1 antibody for immunoprecipitation. Each immunoprecipitate was mixed with 100 μl of the Amplex Red reaction buffer (Amplex Red Phospholipase D assay kit; Thermo Fisher Scientific). The PLD activity was assayed in triplicate for each sample by determining the fluorescence activity after 25 min incubation at 37 °C in the dark with the Multi-Detection Microplate Reader (Berthold, Germany).

### siRNA-mediated gene knockdown

About 2 × 10^5^ HT29 cells were inoculated in a six-well plate and incubated at 37 °C overnight, and then siRNAs were added for 48 h. For knock-down experiments, siATG7 (SignalSilence #6604; Cell Signaling Technology), siBeclin-1 (SignalSilence #6222; Cell Signaling Technology), siAMPK (siAMPK #1: 5′-CGACUAAGCCCAAAUCUUU-3′, siAMPK #2: 5′-ACCAUGAUUGAUGAUGAAGCCUUAA-3′) were used and then cells were treated with 0 or 5 μM concentration of lomitapide at 24 h prior to the endpoint. After lomitapide treatment, the plate was incubated at 5% CO_2_ at 37 °C for 24 h. siTSC2 (SignalSilence #6476; Cell Signaling Technology), siPLD1 (5′-AAGGUGGGACGACAAUGAGCA-3’), siPLD2 (5′-AAGAGGUGGCUGGUGGUGAAG-3′) were used for the same experiment except for 4 h of 0 or 10 μM lomitapide treatment.

### Statistical analysis

All cell experiments were performed in triplicate and the results were expressed as means ± SEM. Differences between the two groups were analyzed by the two-tailed Student’s unpaired *t-*test using GraphPad Prism7. The *n* used in each statistical test is indicated in the figure legends. A *P* value of <0.05 was taken as evidence of statistical significance and is indicated in the figures. (*P < 0.05, **P < 0.01, ***P < 0.001).

## Results

### Lomitapide inhibits mTORC1 in vitro

**Fig. 1:**
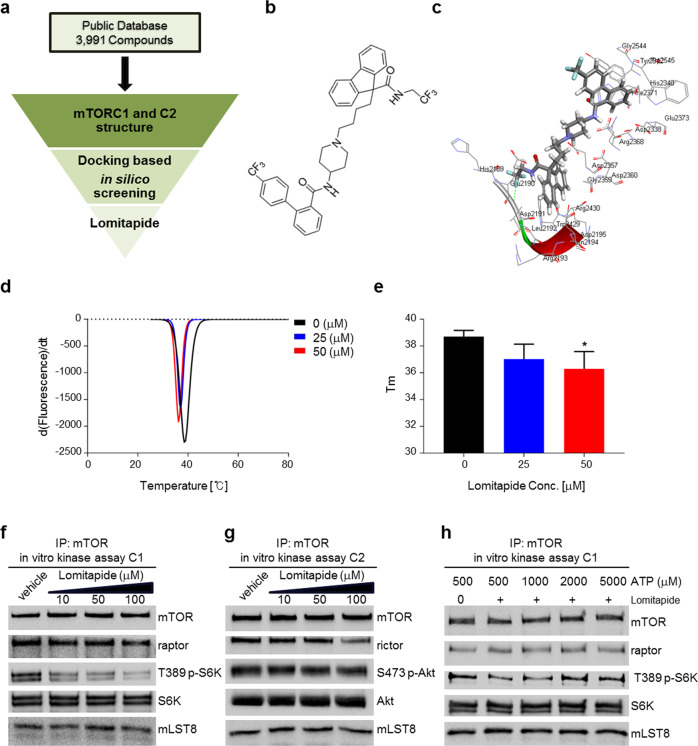
Identification of lomitapide as a mTORC1 inhibitor. **a** Flowchart of in silico virtual screening. **b** Structure of lomitapide. **c** The docking pose of lomitapide within the ATP-binding core of human mTORC1. **d**, **e** The thermal denaturation of recombinant mTOR kinase domain was measured in the absence and presence of varying lomitapide concentrations, as indicated. Representative derivative (dF/dT) curves are shown for untreated and treated with varying ligand concentrations (**d**). Midpoint temperatures of the protein-unfolding transition (Tm) are presented as bars (**e**). Values are mean ± SD of at least three independent measurements (*P < 0.05). **f** In vitro kinase assays were performed in the absence or presence of lomitapide using mTOR immunoprecipitates prepared from HEK293T cell lysates. mTORC1 kinase activity was assessed via immunoblotting of T389 S6K1 phosphorylation. **g** mTORC2 kinase activity was assessed based on S473 Akt phosphorylation. **h** In vitro mTORC1 activity was assayed in the presence of 50 μM lomitapide and increasing concentrations of ATP. T389 phosphorylation of S6K1 was measured via immunoblotting.

To investigate novel anti-neoplastic agents in a cost-effective way, we designed in silico structure-based modeling of mTOR as its druggable potential [38]. We strategized to obtain promising mTOR inhibitors within already approved drugs maximizing therapeutic benefits while avoiding risks of toxicity. In order to repurpose medications from a public database that included FDA-approved drugs, we screened to characterize the interactions of the crystalline structure of human mTORC1 (PDB 4JSV15) [39] (Fig. 1a) with the structures of over 3000 compounds. Of these top-ranked compounds from initial screening, we focused on lomitapide because it fits our criteria of drug repositioning strategy; (i) originally approved not for cancer treatment, (ii) currently used as a cure for orphan (rare) diseases. This compound was first developed to treat a rare genetic disease, hypercholesterolemia, by inhibiting the hepatic MTTP [34, 35, 40] (Fig. 1b). Our docking models revealed that lomitapide specifically binds to the ATP-binding catalytic core of mTORC1, including the H2189, D2190, L2192, Q2194, D2195, D2338, and D2357 residues (Fig. 1c). Interestingly, lomitapide failed to interact with mTORC2 (PDB 5ZCS17) according to the docking analysis, suggesting that there was no interaction between lomitapide and mTORC2.

We tested direct interaction between lomitapide and a purified mTOR kinase domain. The thermal stability of the recombinant mTOR kinase domain was evaluated by varying concentrations of lomitapide. We found that lomitapide reduced the thermal stability of recombinant mTOR kinase domain in a concentration-dependent manner, i.e., 50 µM of lomitapide decreased the Tm by approximately 2.4 °C (Fig. 1d, e), suggesting that lomitapide binds to the kinase domain of mTOR and affects its kinase activity. Next, in vitro mTOR kinase assays were performed using mTOR immunoprecipitates prepared from HEK 293 T cells to determine whether lomitapide directly influences mTOR activity. Lomitapide dose-dependently inhibited the phosphorylation of the T389 residue of S6K1, a major substrate of mTORC1, suggesting its direct role in inhibiting mTORC1 (Fig. 1f). However, this inhibitory effect of lomitapide was not observed in the mTORC2 kinase assay using Akt as the substrate (Fig. 1g). These results indicate that lomitapide selectively inhibits mTOR activity by only inhibiting mTORC1 and not mTORC2. Results of our docking analysis shown in Fig. 1c prompted us to further test the inhibition of lomitapide in competition with ATP. In vitro mTORC1 kinase assays demonstrated that the inhibition of mTORC1 by lomitapide was reversed by increasing ATP concentrations (Fig. 1h), suggesting that lomitapide competes with ATP to directly interact with the kinase domain in order to inhibit mTORC1.

### Lomitapide inhibits cancer cell growth and mTOR signaling

**Fig. 2:**
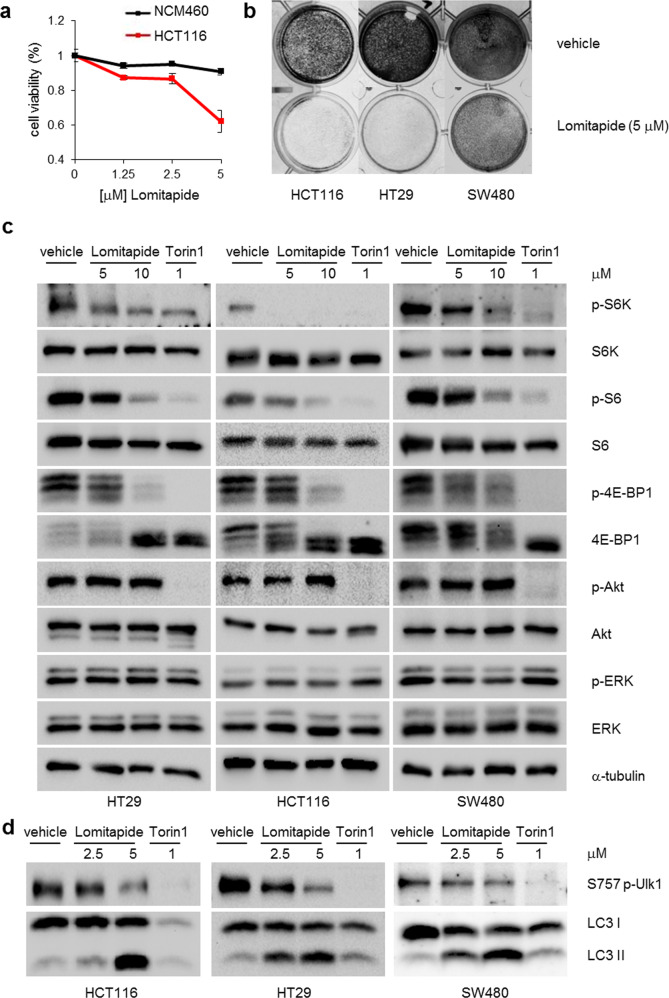
Lomitapide inhibits cancer cell viability and reduces mTOR signaling. **a** Cancer-specific growth inhibition of lomitapide on HCT116 colorectal cancer cell line. **b** Colony formation of vehicle or lomitapide-treated were measured in HT29, HCT116, and SW480 CRC cells. **c** mTOR downstream signaling defects in CRC cells were analyzed by immunoblotting treated with vehicle or lomitapide for 24 h at the indicated concentration. The mTOR inhibitor Torin1 was used as a control. **d** S757 phosphorylation of ULK1 and LC3 levels were measured by immunoblotting to assess autophagy induction.

Next, we examined whether lomitapide substantially impacts cellular growth, which is the primary event that mTOR controls. Lomitapide treatment significantly reduced the viability of multiple colorectal cancer (CRC) cell lines (HCT116, HT29, and SW480), but not of NCM460, a normal human colon mucosal epithelial cell (Fig. 2a and Supplementary Fig. 1). We also found that lomitapide treatment markedly inhibited the colony formation of our CRC cell lines (Fig. 2b). The expression levels of MTTP, lomitapide’s known target, were undetected in our CRC cells, suggesting that the anticancer activity of lomitapide was independent of its known target, MTTP (Supplementary Fig. 2).

In order to investigate the role of lomitapide in cellular mTOR signaling events, CRC cells were treated with lomitapide for 4 h, after which reduced phosphorylation was observed at T389 of S6K1, S240/244 of S6, and T37/46 of 4E-BP1, but not at S473 of Akt nor at T202/Y204 of Erk (Fig. 2c). The phosphorylation levels of PTEN and MEK were unaffected by lomitapide treatment (Supplementary Fig. 3). We further examined whether lomitapide affects other mTOR upstream regulators. In HT29 cells, knockdown of tuberous sclerosis complex 2 (TSC2) or phospholipase D (PLD) failed to interfere with the inhibition of mTORC1 by lomitapide, as demonstrated by the reduced phosphorylation of T389 of S6K1 and S240/244 of S6 (Supplementary Fig. 4A). Furthermore, the activities of PLD were not changed by lomitapide (Supplementary Fig. 4B, C). These results suggest that lomitapide primarily targets mTORC1 in the PI3K/Akt pathway but other signaling components.

As demonstrated by the mTOR kinase assay (Fig. 1f, h), lomitapide selectively and directly inhibits mTORC1 in cells. Expectedly, we found that the levels of the autophagosomal marker LC3-II increased with lomitapide treatment, which decreased the phosphorylation of the mTORC1-sensitive S757 residue of ULK1 (Fig. 2d), suggesting the induction of autophagy by lomitapide-triggered mTORC1 inhibition. In other breast, skin, and stomach cancer cell lines (e.g., MDA-MB-231, MDA-MB-468, A375, A2058, HS-746T, SNU1, and SNU216), lomitapide treatment also led to inhibition of mTORC1 signaling and accompanied LC3-II induction (Supplementary Fig. 5). Further analyses using a panel of 120 different cancer cell lines revealed that lomitapide reduced the viability of all cancer cells (IC50 = 1.5–5 μM) (Supplementary Table 1), revealing its broad-spectrum anticancer effect. On the basis of these data, we conclude that lomitapide inhibits mTORC1 signaling and impairs cancer cell growth and viability.

### Lomitapide induces autophagic cell death

**Fig. 3:**
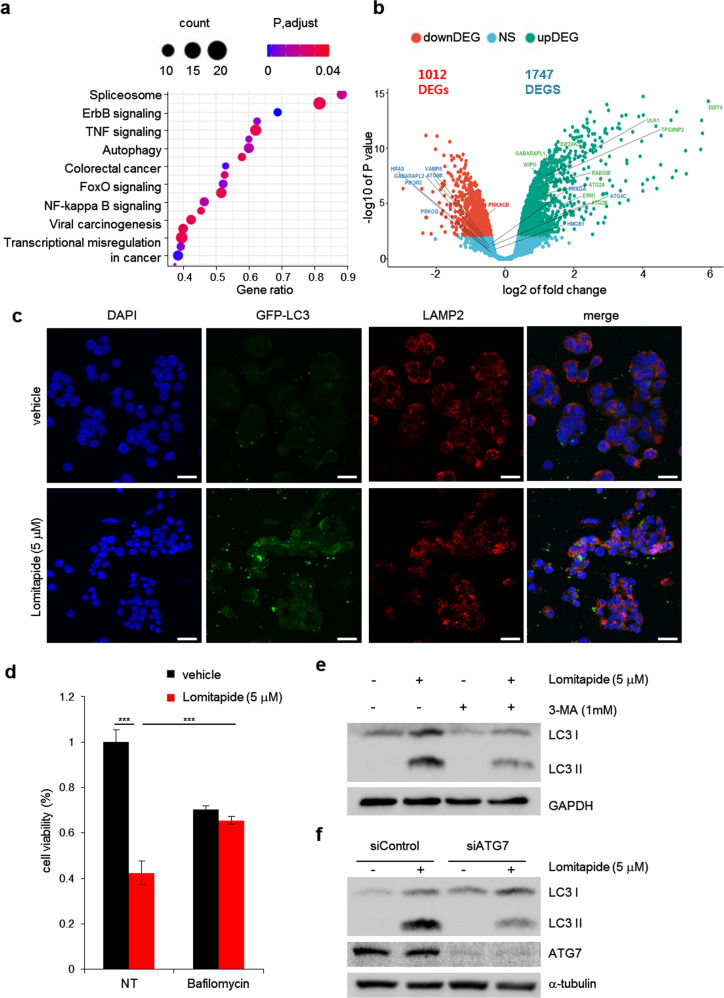
Lomitapide leads to autophagic cancer cell death. **a** Significantly enriched pathways in lomitapide-treated HCT116 cells compared with vehicle-treated cells identified through KEEG analysis. **b** Volcano plot showing significant gene expression changes in response to lomitapide treatment in HCT116 cells. **c** HT29 cells were transfected with GFP-LC3 plasmid for 24 h, and treated with 5 μM lomitapide for another 24 h. GFP-LC3 puncta was visualized by a confocal microscope. Scale bar: 20 μm. **d** Cell viability was measured in HT29 cells treated with 5 μM lomitapide in the absence or presence of 100 nM bafilomycin for 24 h. **e** HT29 cells were treated with 5 μM lomitapide in the absence or presence of 1 mM 3-MA for 24 h. LC3 levels were measured by immunoblotting to assess autophagy induction. **f** si-control and siATG7–transfected HT29 cells were treated with 5 μM lomitapide for 24 h. LC3 levels were measured by immunoblotting to assess autophagy induction.

In order to understand the molecular-level changes in cancer cells treated with lomitapide, we performed RNA-Seq analyses and found that lomitapide significantly impacted the autophagy-related genes, which supported the activation of autophagy by mTOR inhibition (Fig. 3a, b and Supplementary Table 2). To further validate whether the autophagy machinery was triggered by lomitapide treatment, HT29 cells were transfected with green fluorescent protein-LC3 (GFP-LC3), a specific marker of autophagic vesicles and autophagic activity (Fig. 3c). As shown in Fig. 3c, lomitapide treatment significantly increased the number of GFP-LC3 puncta compared with the control group, demonstrating lomitapide-induced autophagy. Similar autophagy induction phenotypes were also observed from lomitapide-treated HCT116 cells (Supplementary Fig. 6A, B). Importantly, reduced cell viability caused by lomitapide treatment was significantly restored when HT29 cells were treated with bafilomycin, a V-ATPase inhibitor that blocks autophagic flux, indicating that lomitapide’s anticancer effect is primarily caused by inducing autophagic cell death (Fig. 3d). HCT116 cell viability was also rescued under 3-methyladenine (3-MA), a class III phosphatidylinositol 3-kinase, treatment (Supplementary Fig. 6C). Inhibition of autophagy by 3-MA was similarly able to protect lomitapide-induced increase of LC3-II in HT29 cells (Fig. 3e) and HCT116 cells (Supplementary Fig. 6D). Knockdown of ATG7 or Beclin-1 was also found to diminish the levels of LC3-II increased by lomitapide treatment in HT29 cells (Fig. 3f) and HCT116 cells (Supplementary Fig. 6E), which validates the lomitapide-mediated activation of autophagy system. During the preparation of this manuscript, Zuo et al. reported that lomitapide plays a role in the control of cancer cell death [41], which is consistent with our findings. These authors reported that lomitapide suppresses the dephosphorylation of AMPK by directly inhibiting protein phosphatase 2 A (PP2A). To test the contribution of AMPK to the autophagy-inducing effect of lomitapide, AMPK levels were depleted by performing an siRNA-mediated AMPK knockdown experiment in HT29 cells (Supplementary Fig. 7). The induction of autophagy triggered by lomitapide was not markedly changed by AMPK depletion (Supplementary Fig. 7), thus demonstrating that lomitapide primarily targets mTOR. We further measured caspase activity to investigate the effect of lomitapide on apoptosis; however, lomitapide-treated CRC cells exhibited negligible induction of caspase 3/7 activities (Supplementary Fig. 8), suggesting no induction of apoptosis. These results thus demonstrate that hyperactivation of autophagy is the prime event underlying lomitapide-triggered cancer cell death.

mTOR inhibitors (e.g., rapamycin, second-generation mTOR inhibitors) have been approved for the treatment of several types of cancer and more of them are being actively tested in clinical trials [42, 43]. Nevertheless, the overall success of rapamycin is limited due to the incomplete inhibition of mTORC1-mediated phosphorylation of 4E-BPs [44, 45]. Moreover, increased eukaryotic translation initiation factor (eIF4E) overexpression renders cancer cells resistant to mTOR inhibitors [46, 47]. An HT29 cell line expressing eIF4E was used to compare the effects of lomitapide and other mTOR inhibitors. The phosphorylation of 4E-BP1 was almost fully abolished by lomitapide, whereas rapamycin only partially inhibited 4E-BP1 phosphorylation (Supplementary Fig. 9A). Compared to rapamycin and PP242, lomitapide also led to the robust induction of LC3-II levels in both control and elF4E-overexpressing HT29 cells (Supplementary Fig. 9A). Importantly, lomitapide suppressed the viability of both control and elF4E-overexpressing HT29 cells, whereas the anticancer effects of rapamycin, PP242, and Torin1 were reduced in elF4E-overexpressing HT29 cells (Supplementary Fig. 9B, C). These results clearly suggest that lomitapide could potentially overcome the limitations of other mTOR inhibitors due to its potent autophagy-inducing properties.

### Lomitapide inhibits the growth of tumor xenografts

**Fig. 4:**
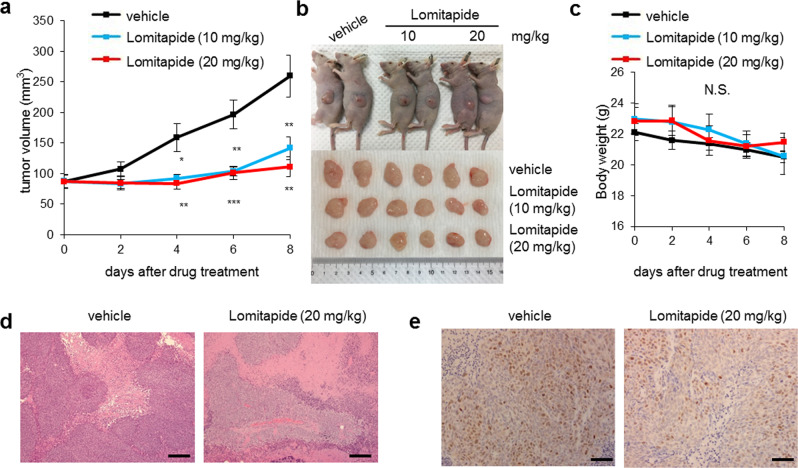
Lomitapide suppresses the growth of tumor xenografts. **a** HT29 cells were inoculated into flanks of nude mice and tumor volumes were measured for 10 days after intraperitoneal injection into xenograft tumors every 2 days. **b** Representative images of xenograft tumors at the day of sacrifice. **c** Body weight of xenograft mice bearing HT29 tumors during the in vivo experiment. Data were expressed as means ± SEM (*P < 0.05; **P < 0.01; ***P < 0.001, Student’s *t*-test). (*n* = 6 per group). **d** Hematoxylin and eosin (H&E) staining of tumor tissues collected from either vehicle or lomitapide-treated mice. **e** Tumor tissues were subjected to immunohistochemistry staining with Ki67 antibodies. Scale bar: 20 μm.

After in vitro studies using cancer cell lines, the effects of lomitapide on tumor growth were examined in vivo by injecting CRC cells subcutaneously into immunocompromised mice and then monitoring tumor growth. The growth of both HT29 and HCT116 CRC xenografts was markedly inhibited by lomitapide treatment (Fig. 4a, b and Supplementary Fig. 10) confirming its anticancer effects in vivo. Lomitapide treatment did not influence body weight, indicating any apparent toxicity (Fig. 4c). Hematoxylin and eosin (H&E) staining of tumor tissues of lomitapide-treated groups showed more neoplastic lesions compared to vehicle groups (Fig. 4d). Through immunohistochemical staining, we further observed that the expression of Ki67 in lomitapide-treated HT29 xenografts was lowered (Fig. 4e). Taken together, these results suggest the therapeutic value and safety of lomitapide as an anticancer agent.

### Lomitapide inhibits the growth of patient-derived CRC organoids

**Fig. 5:**
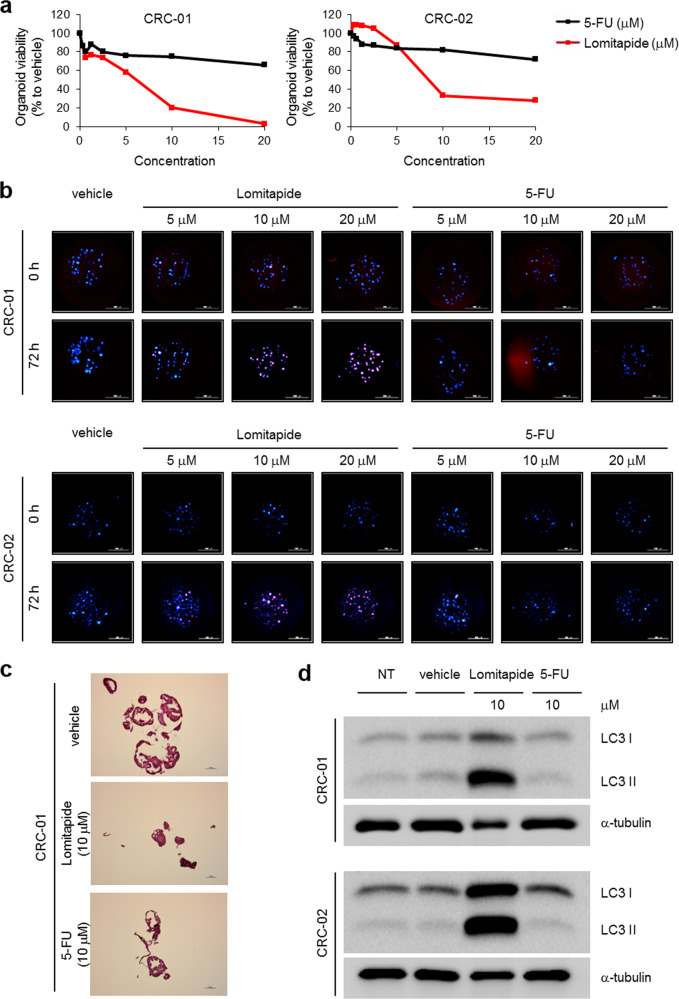
Lomitapide suppresses the growth of patient-derived CRC organoids. **a** Dose-response curves of patient-derived CRC organoids CRC-01 (KRAS^WT^; APC and TP53 mutant) and CRC-02 (KRAS^G12V^; APC and TP53 mutant) treated with 10 μM 5-FU or 10 μM lomitapide. The organoid size was measured and quantified at 48 h of either 5-FU or lomitapide treatment relative to vehicle control. **b** Dose-response images of patient-derived CRC organoids CRC-01 and CRC-02 treated with DMSO, lomitapide, or 5-FU for 72 h at indicated concentrations. Organoids were stained with CFSE as an organoid marker (blue) and PI as a dead cell marker (red). Scale bars: 2 mm for CRC-01 and 1 mm for CRC-02. **c** H&E staining of the original matrigel CRC-01 organoid culture. Scale bar: 1 mm. **d** LC3 levels were measured by immunoblotting to assess autophagy induction. Lysates were prepared from organoids treated with vehicle, 10 μM lomitapide, or 10 μM 5-FU for 48 h.

We next investigated whether the lomitapide’s inhibitory effect could also be confirmed in human tumor organoids that are three-dimensional ex-vivo models having the advantage of retaining the characteristics of the cancer cells from the original patients. When two different patient-derived CRC organoid lines were treated with 10 μM lomitapide, organoid viability as measured from the size of live organoid cells was markedly reduced (Fig. 5a). Importantly, the organoid viability of lomitapide-treated organoids was dramatically reduced compared to that of the organoids treated with 10 μM 5-fluorouracil (5-FU), a first-line chemotherapeutic drug for CRC (Fig. 5a). Seventy-two hours of treatment of lomitapide dose-dependently increased propidium iodide (PI)-stained dead cells in CRC organoids, whereas 5-FU treatment showed only a modest impact on cell viability (Fig. 5b), validating the potent anticancer action of lomitapide. H&E staining further showed a substantial decrease in tumor organoid size in response to lomitapide but not vehicle (Fig. 5c). LC3-II levels were robustly increased by treatment with lomitapide but not with 5-FU (Fig. 5d). Consistent with findings from cancer cell lines and tumor xenografts, these results based on cancer organoid models validate that lomitapide is a potent antitumor drug to trigger autophagic cancer cell death.

### Lomitapide enhances the therapeutic effect of anti-PD-1

**Fig. 6:**
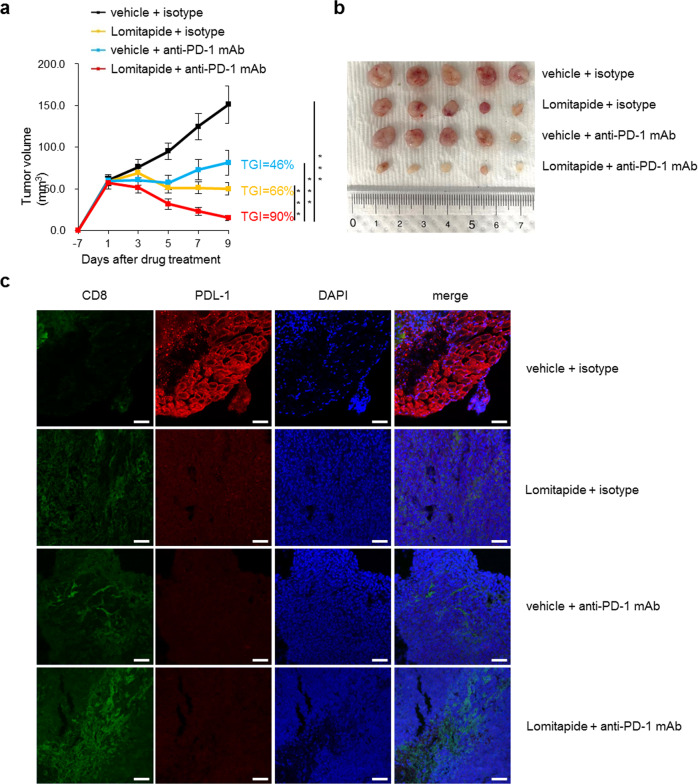
Treatment of tumor-bearing mice with lomitapide improves the therapeutic benefit of anti-PD-1 immunotherapy. **a** Tumor growth curves of MC38 mouse colon cancer cells in mice treated with control (vehicle) or lomitapide combined with either control isotype or anti-PD-1. Lomitapide at 20 mg/kg every day, anti-PD-1 or isotype IgG 10 mg/kg every other day. Statistically significant differences (indicated by asterisks) are calculated using an unpaired two-tailed Student’s *t*-test (*P < 0.05, **P < 0.005, and ***P < 0.0005). **b** Representative images of tumor tissues at 18 days following inoculation of MC38 cells. **c** Representative results of immunofluorescence staining of CD8 (green), PD-L1 (red), and DAPI (blue) in MC38 tumor tissues. Scale bar: 20 μm.

Targeting antibodies to programmed cell death protein-1 (PD-1) is an effective treatment for various cancer types [48, 49]. Although some patients receiving anti-PD-1 therapies respond favorably, most patients undergo disease progression without any clinical benefit; [50–52] this highlights the importance of combining therapies that enhance antitumor immunity. Recently, mTOR inhibitors in combination with anti-PD-1 have been reported to provide more durable and synergistic tumor regression than that by either agent alone [53, 54]. Therefore, we assessed the impact of this combined treatment of antibody-mediated PD-1 blockade along with lomitapide on tumor growth, thereby determining whether it could improve the responsiveness to anti-PD-1 therapy. Our results showed that lomitapide treatment alone decreased the tumor growth in two syngeneic murine models, mouse colon cancer MC38 and melanoma B16-F10 models respectively (Fig. 6a, b and Supplementary Fig. 11A, B). Importantly, the combined treatment with lomitapide and anti-PD-1 antibody significantly inhibited tumor growth compared to anti-PD-1 antibody treatment alone, in both MC38 and B16-F10 tumor models (Fig. 6a, b and Supplementary Fig. 11A, B). Administration of 20 mg/kg of lomitapide resulted in no apparent changes in the body weight of mice (Supplementary Fig. 12A) and no toxicity in the liver, kidney, and lung tissues (Supplementary Fig. 12B). Immunohistochemical staining for tumor tissue sections revealed that the combination of anti-PD-1 antibody and lomitapide significantly increased the infiltration of CD8^+^ T cell populations into the tumor (Fig. 6c and Supplementary Fig. 11C). Collectively, our results provided strong evidence that combining lomitapide makes tumor-bearing mice responders to anti-PD-1 therapy.

## Discussion

Upregulated mTOR signaling activities and hyperactive *MTOR* mutations have been reported in various types of cancer [27, 29, 30]. Therefore, it is essential to discover therapeutic interventions that can inhibit mTOR actions. In this study, we performed in silico screening of mTOR-binding compounds and identified lomitapide, an FDA-approved drug, as a candidate to inhibit mTOR and its signaling in cancer cell growth. In vitro characterization of lomitapide’s inhibition of mTOR and analysis of its impact in cancer cells demonstrates lomitapide’s inhibition of mTORC1. Suppression of mTORC1 signaling in lomitapide-treated cancer cells and human CRC organoids triggers a robust induction of autophagy, which mainly drives lomitapide-mediated cancer cell death. The inhibitory effect of lomitapide on cancer cell growth was also validated in vivo using tumor xenograft models. Furthermore, combining lomitapide treatment significantly enhances the efficacy of anti-PD-1 therapy in reducing the growth of tumors in CRC and melanoma; this establishes efficacious anticancer effects of lomitapide across multiple different cancer types in preclinical in vivo models. Therefore, our drug repurposing strategy, starting from virtual screening to validation and from mechanistic analyses to in vivo characterizations, represents an illustrative model that can be potentially valuable for the next generation of translational medicine.

Lomitapide was originally approved by the FDA for therapeutic use to treat homozygous familial hypercholesterolemia, a serious rare inherited medical condition that leads to extremely high levels of low-density lipoprotein cholesterol [34, 35]. Mechanistically, lomitapide acts in the liver by inhibiting microsomal triglyceride transfer protein (MTTP) that is required to assemble the low-density lipoprotein particle [55]. We completely ruled out the possibility that lomitapide’s anticancer effects might be mediated by its action on MTTP. There was no data supporting the proto-oncogenic role of MTTP, which is expressed in the liver and the intestine. Conditional deletion of hepatic MTTP in mice has been linked to alterations in liver metabolism, but not to hepatic cellular growth defects [56]. Intestine-specific knockout of murine MTTP rather increased the tumor burden in a colitis-associated carcinogenesis model [57]. Importantly, we observed no notable expression of MTTP in CRC cell lines examined in this study. Therefore, we suggest that the anticancer effects of lomitapide in cancer cells strongly implicate its engagement of mTOR and not MTTP.

Collectively, our findings elucidate that lomitapide-mediated inhibition of mTORC1 signaling leads to the autophagic death of cancer cells. Lomitapide-treated cancer cells exhibited a robust induction of LC3-II with no signs of activated apoptosis (caspase 3/7). Pharmacological intervention of autophagy further reduced the already lowered cancer cell viability by lomitapide treatment, validating autophagic cell death as the primary mechanism of lomitapide’s anticancer effects. As a potent mTORC1-inhibiting autophagy inducer, lomitapide appears to overcome the limitations of other mTOR inhibitors. Despite its potential in cancer treatment, first-generation allosteric mTOR inhibitors (including rapamycin) have shown some success in specific tumor types but have not exhibited broad anticancer activity due to the feedback activation of PI3K–PKB signaling and incomplete dephosphorylation of 4E-BPs [44, 46, 58]. Given that lomitapide acts as an ATP-competitive mTOR kinase inhibitor, lomitapide showed robust inhibition on 4E-BP1 phosphorylation. Importantly, in the present study, unlike other ATP-competitive, second-generation mTOR inhibitors, lomitapide was shown to exert anticancer effects on cancer cells with eIF4E overexpression. These findings support the therapeutic advantages of lomitapide over other mTOR inhibitors. In light of our results and Zuo et al.’s report [41], we believe that lomitapide’s superior anticancer activity can be achieved by targeting multiple autophagy signaling hubs including mTORC1 and PP2A.

Autophagy is a physiological homeostatic process involved in cellular protection through the degradation of aged and misfolded proteins as well as damaged organelles such as mitochondria. In normal cells, amino acid deprivation or glucose depletion is a canonical signal to inactive mTORC1 to turn on, thereby elevating autophagic fluxes. When induced in excess, autophagy can result in cancer cell death by intensifying self-digesting autophagosomal activities. Our results demonstrate that the contribution of this hyperactive autophagy is critical in determining the ultimate fate of cancer cells. In addition to mTOR inhibitors, other autophagy-inducing drugs have been identified to treat cancer [16–19]. For example, gossypol, a pan-Bcl-2 inhibitor, was reported to trigger autophagic cell death in malignant gliomas [59]. Notably, both lomitapide-triggered autophagic induction and tumor growth suppression were consistently detected in patient-derived CRC organoid cultures, which reflect the heterogeneous nature of CRC. Compared to 5-FU, a first-line chemotherapeutic treatment for CRC, lomitapide exhibited autophagy activation with potent suppression of tumor organoid growth, signifying its therapeutic value as an anticancer drug. Although the results presented here are encouraging, the exact mechanism by which lomitapide influences cellular mTORC1 but not mTORC2 still needs to be understood. Furthermore, whether mTORC1 inhibition is the only way or one of several ways by which lomitapide induces autophagy still remains to be investigated. Considering contexts wherein autophagy can be utilized as a survival strategy to promote cancer cell survival, the therapeutic conditions of lomitapide in should be further refined. However, taken together, our results suggest the potential clinical use of lomitapide to treat these devastating tumors.

As with conventional cancer therapies, strategies to enhance clinical responses with immune checkpoint blockade are being investigated to provide breakthroughs in clinical immuno-oncology. Therefore, extensive pharmacological and clinical trials are currently underway to determine the safety and efficacy of combining current immune checkpoint drugs with conventional cancer treatments and immunomodulatory agents. We demonstrated that lomitapide markedly improves antitumor responses conferred by anti-PD-1 administration in syngeneic colon cancer MC38 and melanoma B16-F10 models, thereby potentially extending the scope for its potential use in combination with other therapies. Lomitapide alone also caused a comparable suppression of tumor growth in these models; therefore, we speculate that lomitapide-induced cancer cell death increases neoantigen production to stimulate an antitumor immune response in the tumor microenvironment. Similar to the results of previous studies using mTOR inhibitors, PD-L1 expression in cancer cells could be reduced by lomitapide treatment, suggesting its tumor-intrinsic effects. Modulating the tumor-extrinsic activities of mTOR signaling has also been known to determine the antitumor response induced by anti-PD-1 therapy. Typically, mTOR inhibition (e.g., rapamycin and rapalogs) has been understood to elicit immunosuppression; however, recent reports indicate potential immune-boosting functions following the pharmacological or genetic ablation of mTOR signaling pathways [53, 54]. For instance, induction of CD8 memory T cell formation or reduction of myeloid-derived suppressor cells appears to be controlled by mTOR inhibition [60, 61]. Following our discovery of lomitapide as a candidate for synergistic combination therapy, additional studies are required to evaluate the additional impact of lomitapide on shaping host immunity in the tumor microenvironment.

Collectively, our findings demonstrate that structure-based drug discovery approaches can be used to identify potential drugs to inhibit mTOR signaling. Based on multiple lines of evidence, we reveal that the lipid-lowering drug, lomitapide, possesses an autophagy-mediated anti-tumoral effect through mTOR pathway regulation. Lomitapide has already been established as a safe drug to treat familial hypercholesterolemia in humans, as long-term administration of lomitapide resulted in no complications such as spontaneous cancer development [62]. Its side effect profile is generally more favorable than that of most drugs typically used to treat cancer. These results highlight the importance of conducting clinical investigations to assess the use of lomitapide to treat cancer patients.

## Supplementary information

Supplementary Information

Original Data File

Reproducibility checklist

## Data Availability

All data needed to evaluate the conclusions in the paper are present in the paper and/or the Supplementary information. All other data are available from the corresponding author upon reasonable request.
