# Supplementary material for: Lomitapide, a cholesterol-lowering drug, is an anticancer agent that induces autophagic cell death via inhibiting mTOR
Source: Cell Death Dis. 2022 Jul 12;13(7):603. doi: 10.1038/s41419-022-05039-6 (PMC9279289; doi:10.1038/s41419-022-05039-6)
Supplement: Supplementary file 1 — Supplementary Information [file 41419_2022_5039_MOESM1_ESM.docx]

**Supplementary information**

**Supplementary Fig. 1**

**
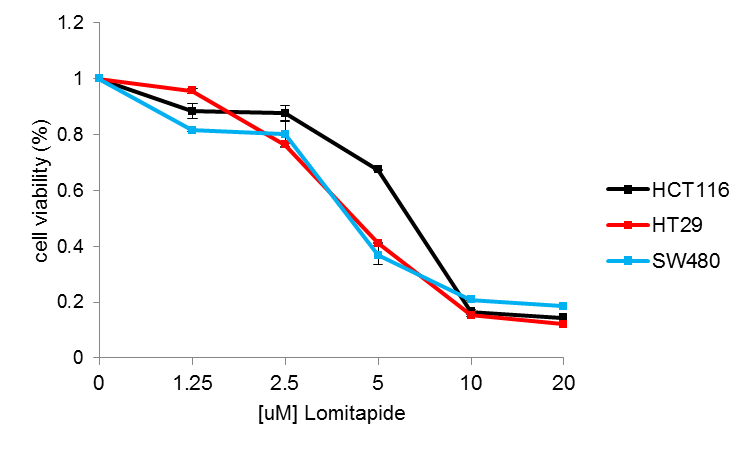
**

**Supplementary Fig. S1**

HCT116, HT29, and SW480 CRC cells were treated with lomitapide for 24 h and cell viability was measured.

**Supplementary Fig. 2**


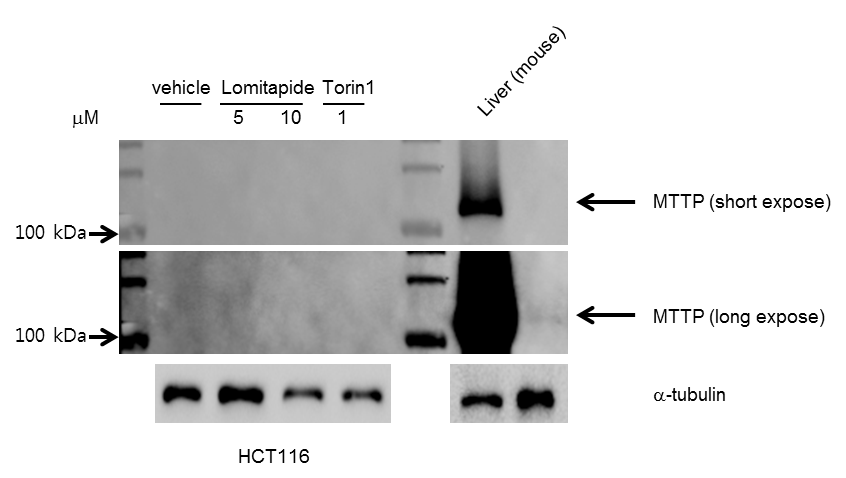


**Supplementary Fig. S2**

Expression of MTTP in HCT116 CRC cells and mouse tissue lysates (liver and colon) was measured by immunoblotting.

**Supplementary Fig. 3**


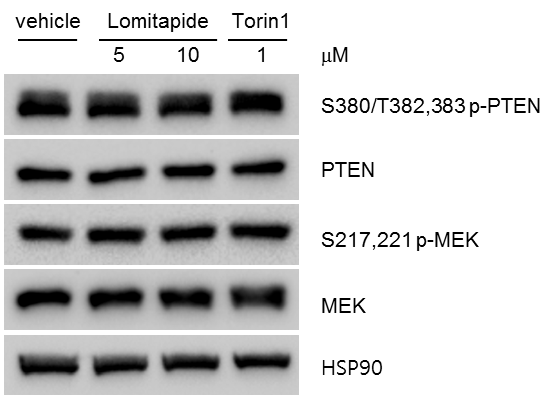


**Supplementary Fig. S3**

HT29 cells were treated with vehicle, lomitapide, or Torin1 for 4 h at indicated concentration. Expression and phosphorylation of PTEN and MEK proteins were analyzed by immunoblotting.

**Supplementary Fig. 4**


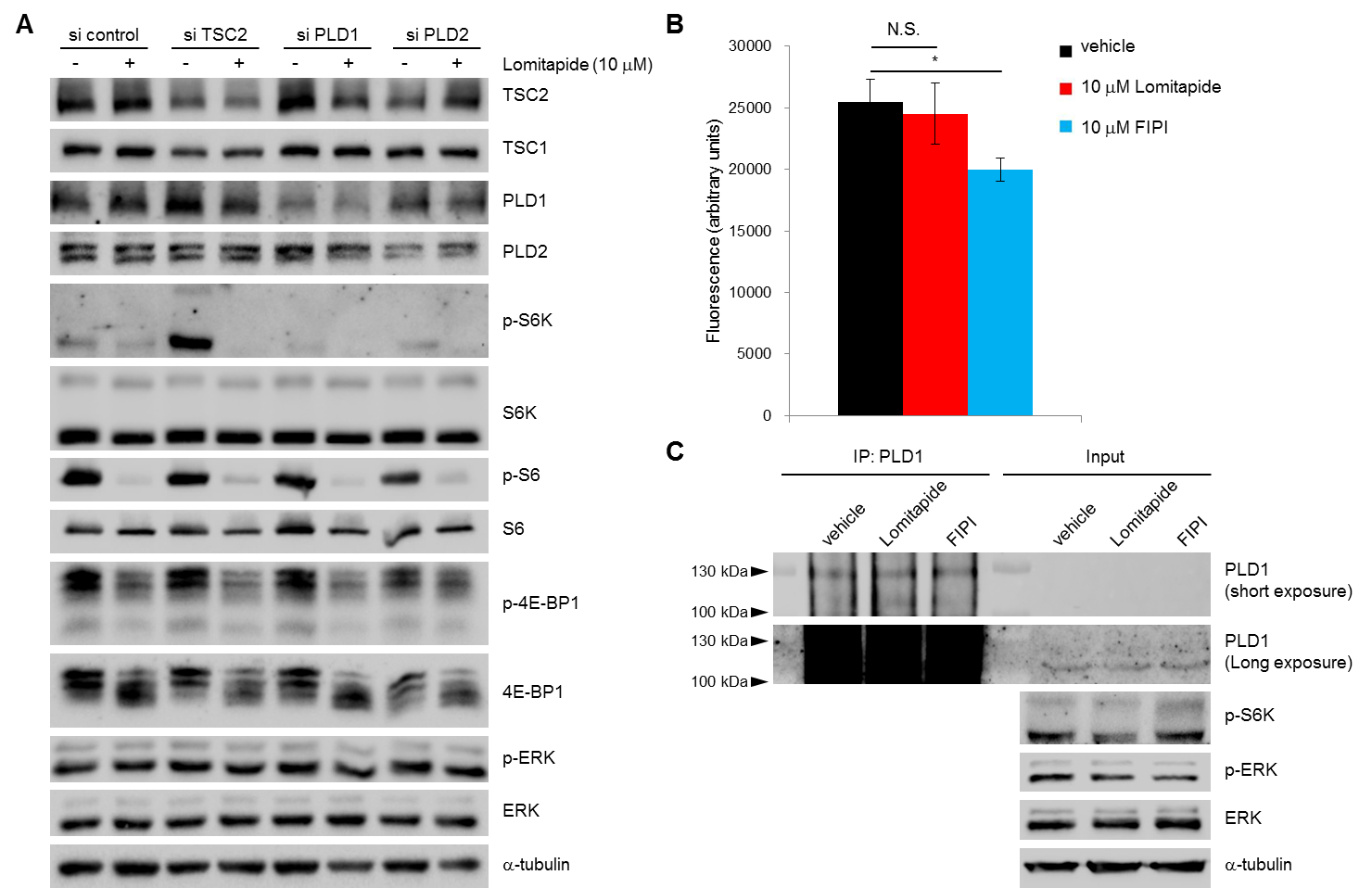


**Supplementary Fig. S4**

**A** si-control, si-TSC2, si-PLD1, or si-PLD2–transfected HT29 cells were treated with 10 μM lomitapide for 4 h. Total and phosphorylation levels of proteins were measured by immunoblotting to assess mTOR signaling. **B**, **C**, PLD activities were measured from HT29 cells treated with vehicle, lomitapide, or the PLD inhibitor FIPI for 4 h. (**B**) Amounts of PLD1 in immunoprecipitates and levels of protein phosphorylation were analyzed by immunoblotting (**C**).

**Supplementary Fig. 5**


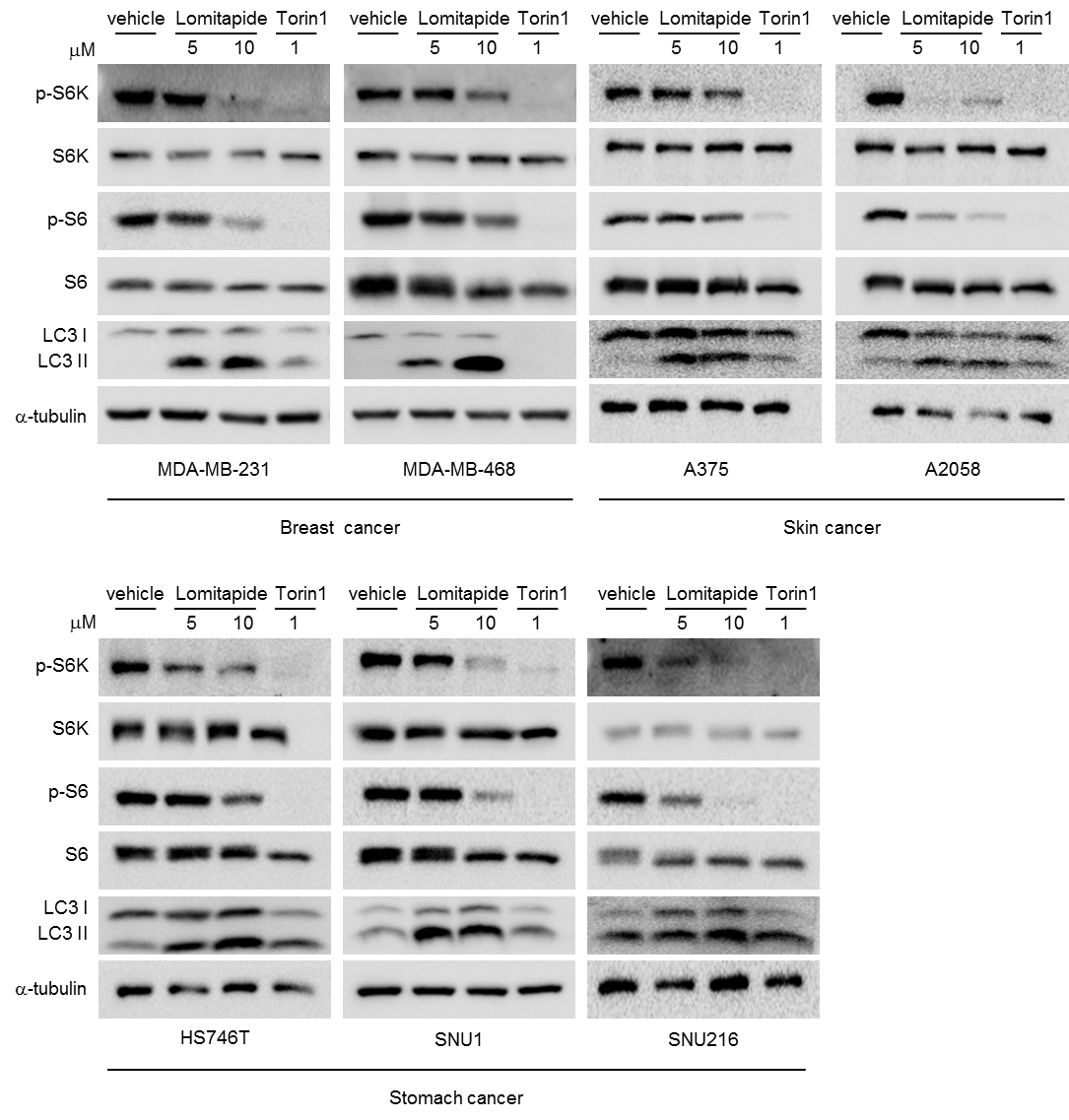


**Supplementary Fig. S5**

mTORC1 signaling changes and autophagy induction in various human cancer cells were measured by immunoblotting. mTORC1 signaling and autophagy induction at 4 h and 24 h of lomitapide treatment respectively at indicated concentrations.

**Supplementary Fig. 6**

**
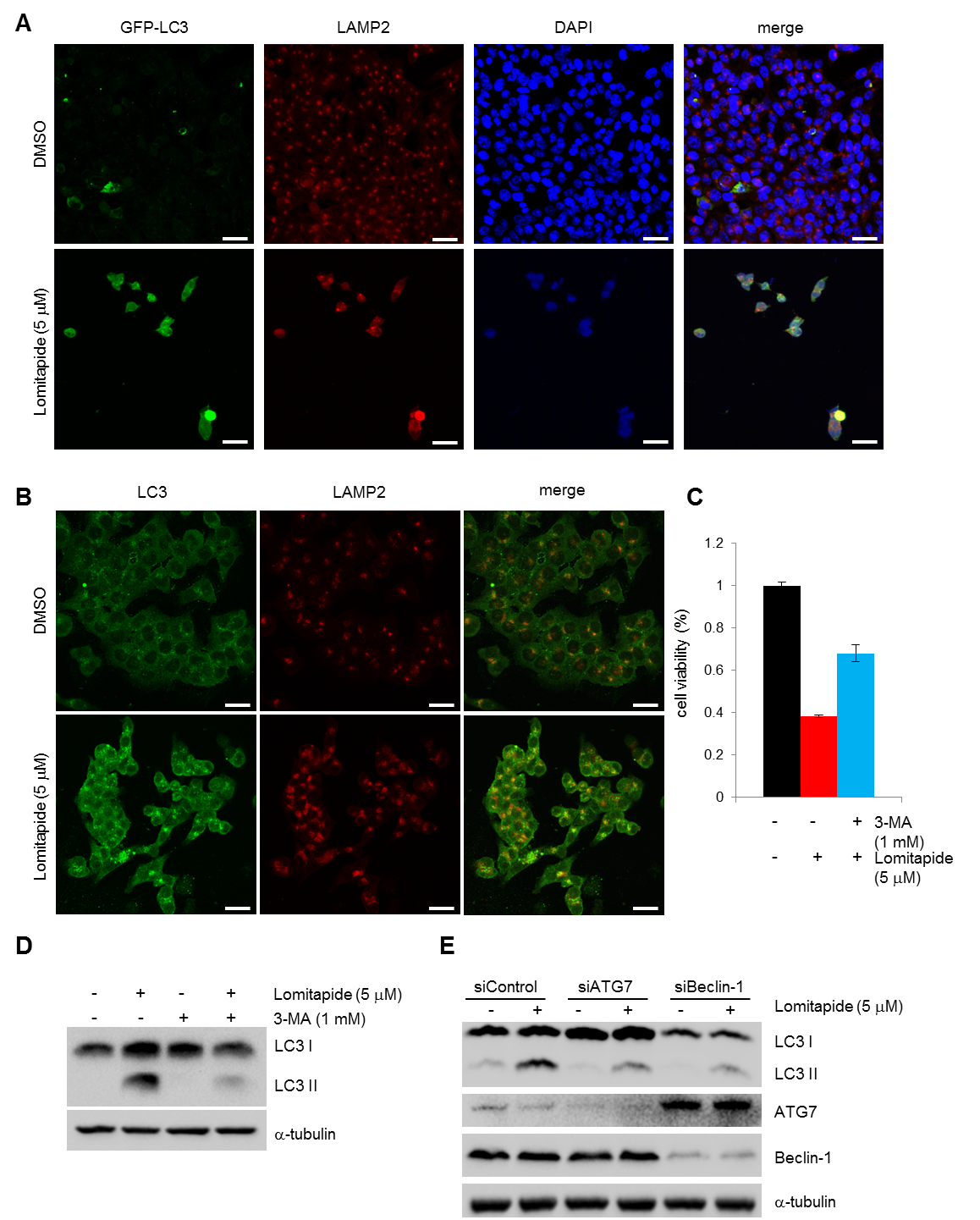
**

**Supplementary Fig. S6**

**A** HCT116 cells were transfected with GFP-LC3 plasmid for 24 h, and treated with 5 μM lomitapide for another 24 h. GFP-LC3 puncta was visualized by confocal microscope. Scale bar: 20 μm. **B** Endogenous LC3B level was detected in HCT116 cells by anti-LC3B antibody under 5 μM lomitapide for 24 h treatment. Scale bar: 20 μm. **C** Cell viability was measured in HCT116 cells treated with 5 μM lomitapide in the absence or presence of 1 mM 3-MA for 24 h. **D** LC3 levels were measured by immunoblotting to assess autophagy induction. HCT116 cells treated with 5 μM lomitapide in the absence or presence of 1 mM 3-MA for 24 h. **E** si-control, si-ATG7, and si-Beclin-1–transfected HCT116 cells were treated with 5 μM lomitapide for 24 h. LC3 levels were measured by immunoblotting to assess autophagy induction.

**Supplementary Fig. 7**


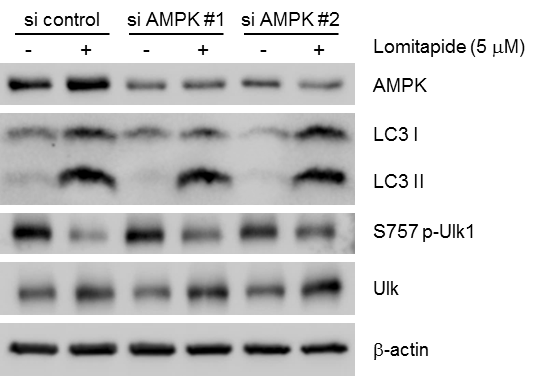


**Supplementary Fig. S7**

si-control or si-AMPK–transfected HT29 cells were treated with 5 μM lomitapide for 24 h. LC3 levels were measured by immunoblotting to assess autophagy induction. Total and phosphorylation levels of Ulk1 were measured by immunoblotting to validate mTORC1 suppression.

**Supplementary Fig. 8**


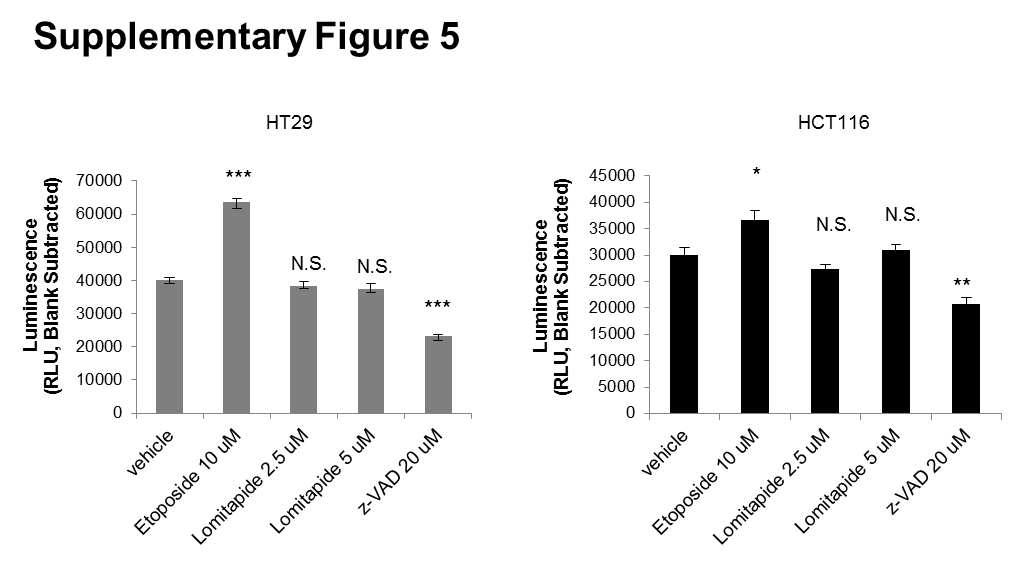


**Supplementary Fig. S8**

Caspase activity was measured from HT29 and HCT116 cells treated with lomitapide. etoposide, and z-VAD for 24 h. Data are expressed as means ± SEM (**P* < 0.05; ***P* < 0.01; ****P* < 0.001, Student’s *t* test). Etoposide was used as control for the induction of apoptosis. z-VAD was used as control for the inhibition of apoptosis.

**Supplementary Fig. 9**

**
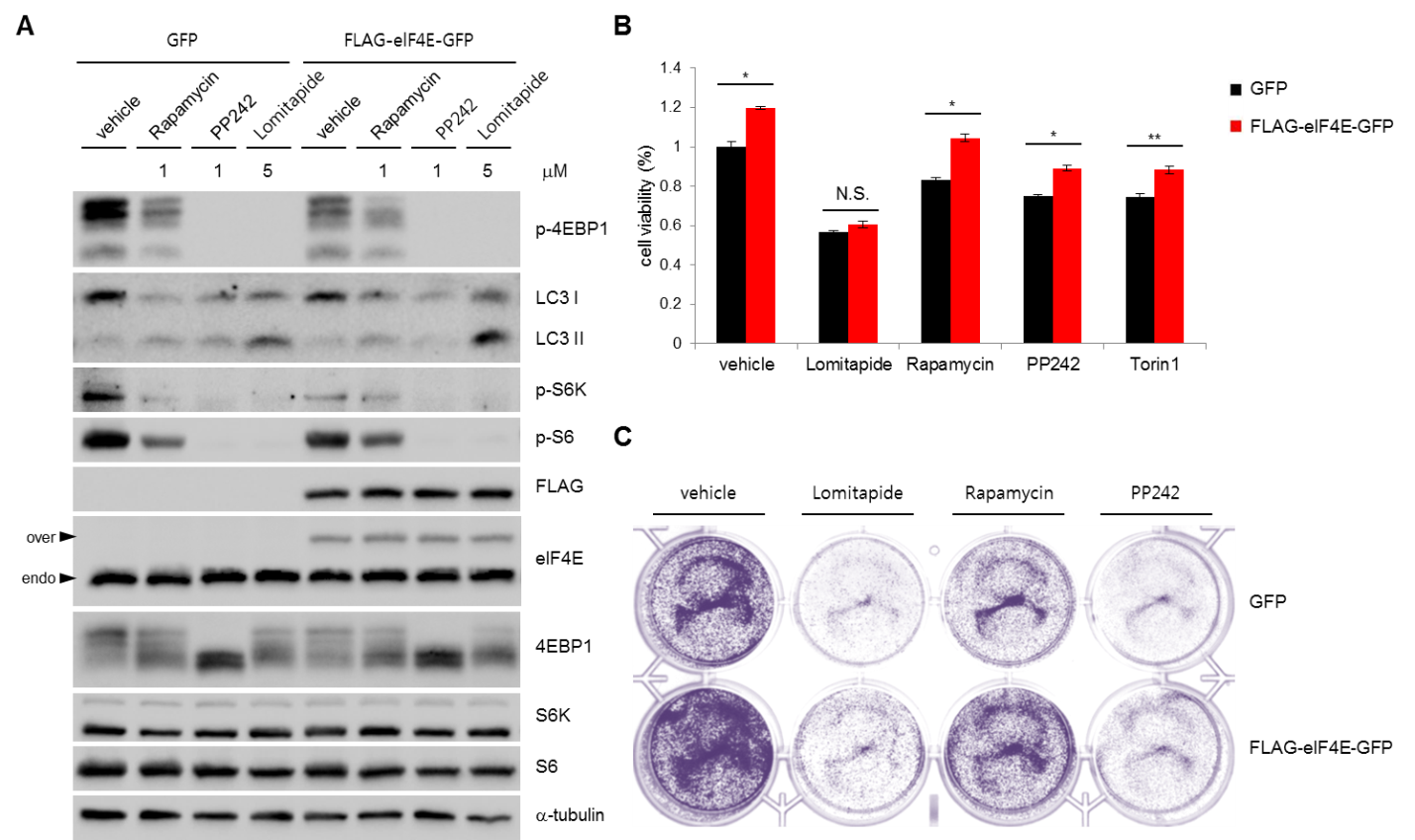
**

**Supplementary Fig. S9**

**A** HT29 cell line stably expressing either GFP or FLAG-eIF4E were treated with vehicle, rapamycin, PP242, or lomitapide for 24 h at indicated concentrations. Levels of signaling proteins and their phosphorylation were measured by immunoblotting. **B**, **C**, MTT assay were measured from eIF4E-overexpressing HT29 cells treated with vehicle, lomitapide, rapamycin, PP242, or Torin1 for 48 h (**B**). Colony formation was analyzed under 96 h-treatment conditions (2 μm each) (**C**). Data are expressed as means ± SEM (**P* < 0.05; ***P* < 0.01, Student’s *t* test).

**Supplementary Fig. 10**

**
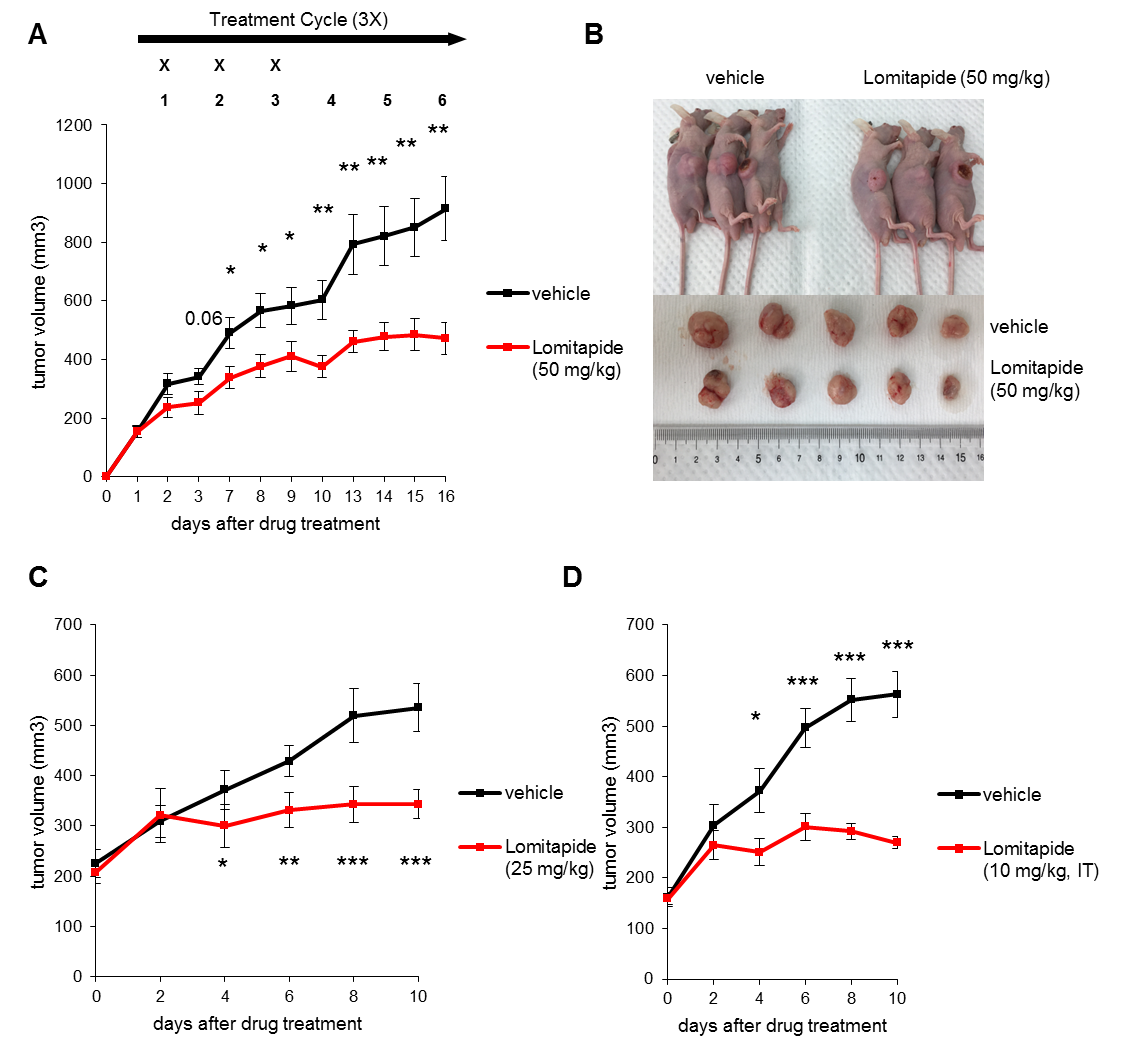
**

**Supplementary Fig. S10**

**A**, **B**, Nude mice bearing HCT116 xenografts were intraperitoneally treated with lomitapide (50 mg/kg) or vehicle. Tumor volumes were calculated and presented as growth curves (**A**). Representative tumor tissues resected from mice on day 18 after treatment (**B**). **C**, **D**, HCT116 cells were inoculated into flanks of nude mice (n = 6 per group) and tumor volumes were measured for 10 days after intraperitoneal (**C**) or intratumoral (**D**) injection of lomitapide as indicated every 2 days. Data are expressed as means ± SEM (**P* < 0.05; ***P* < 0.01; ****P* < 0.001, Student’s *t* test).

**Supplementary Fig. 11**

**
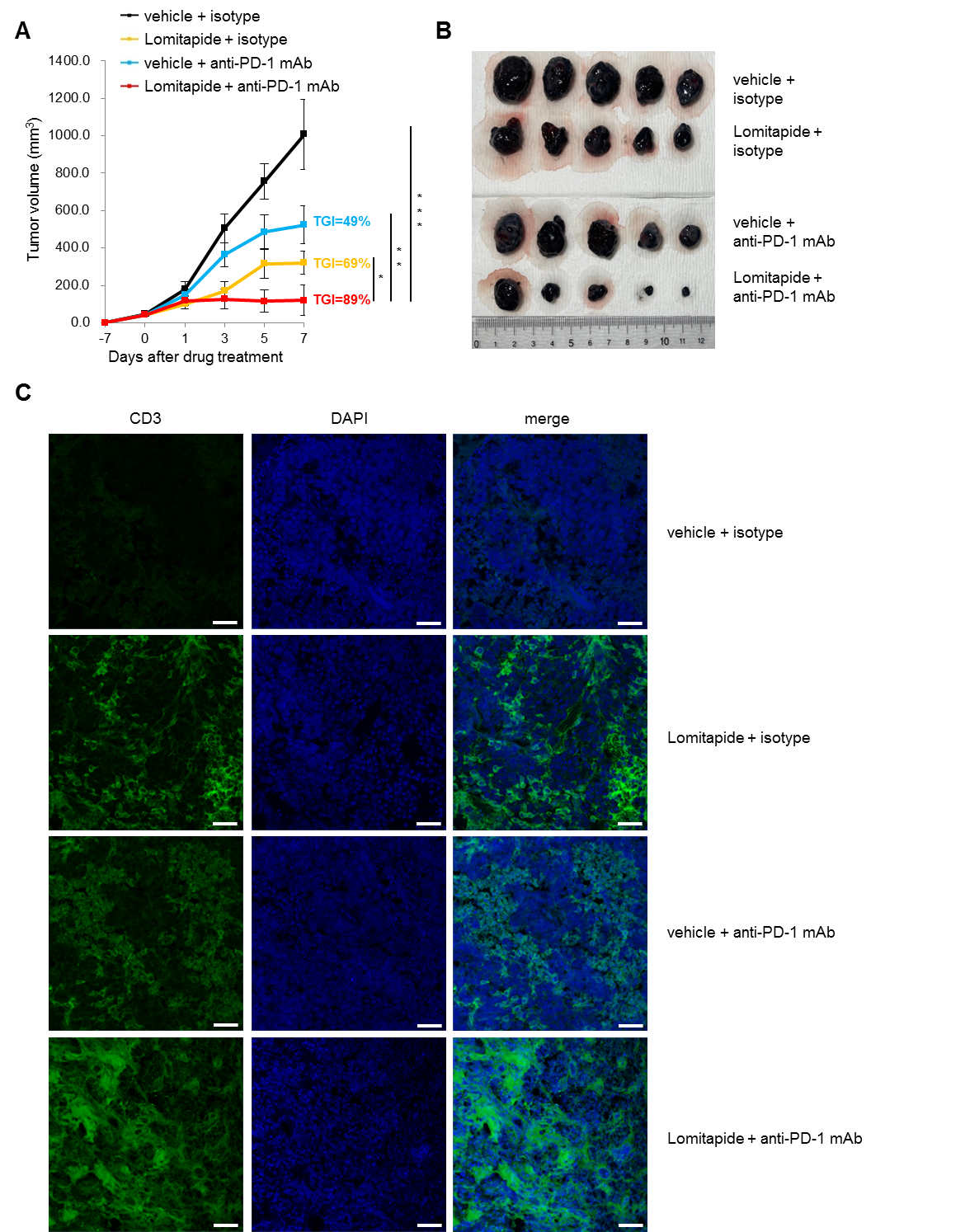
**

**Supplementary Fig. S11**

**A** Tumor growth curves of B16F10 mouse melanoma cells in mice treated with control (vehicle) or lomitapide combined with either control isotype or anti–PD-1. Lomitapide at 20 mg/kg every other day, anti PD-1 or isotype IgG at 7.5 mg/kg were administered on days 1, 4, 7, and 10 in PBS from 10 days after innoculation. Statistically significant differences (indicated by asterisks) are calculated using an unpaired two-tailed Student’s *t* test (**P* < 0.05, ***P* < 0.005, and ****P* < 0.0005). **B** Representative images of tumor tissues at 15 days following inoculation of B16F10 cells. **C** Representative images of immunofluorescence staining of CD3 (green), and DAPI (blue) in B16F10 tumor tissues. Scale bar: 20 μm.

**Supplementary Fig. 12**

**
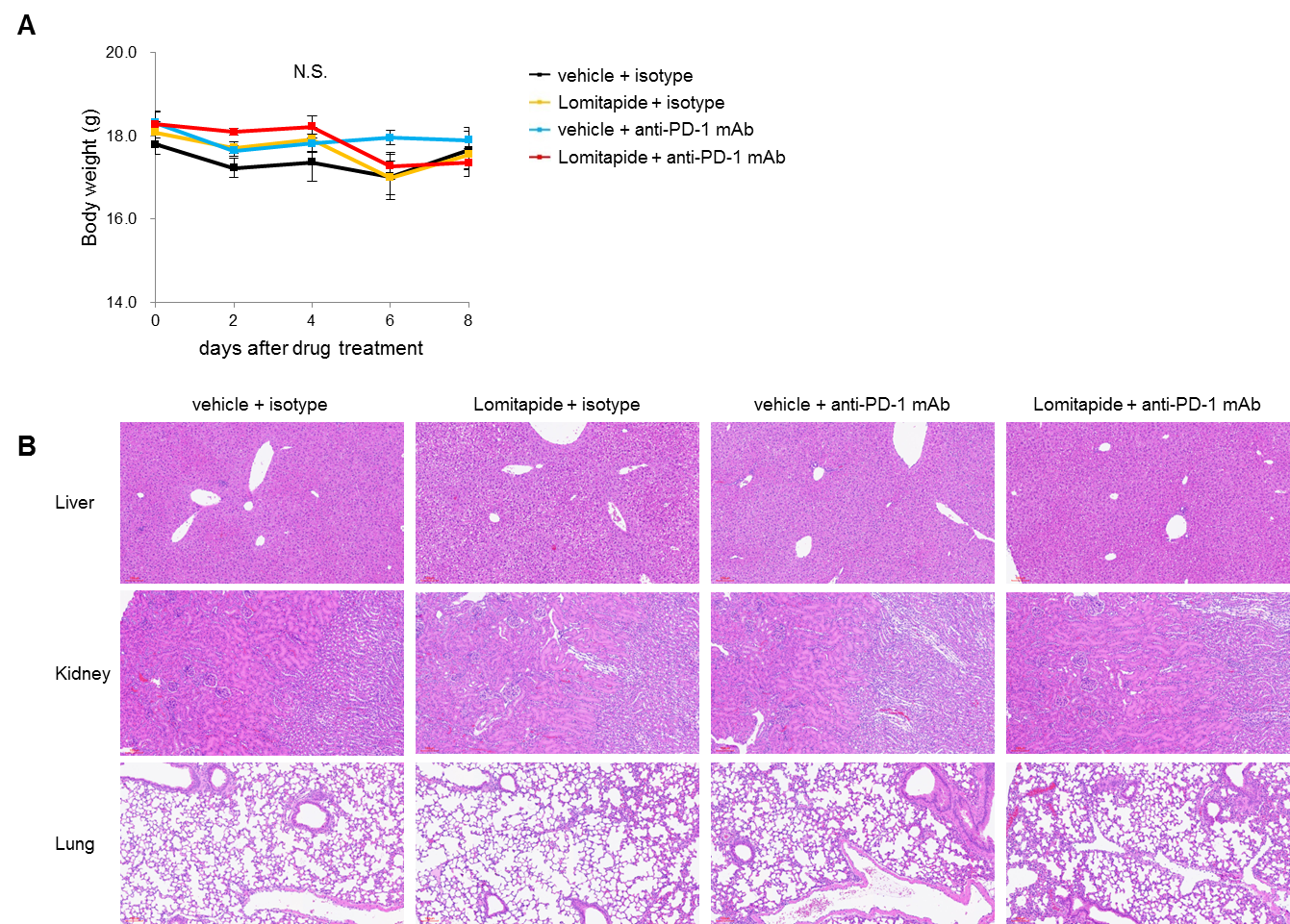
**

**Supplementary Fig. S12**

**A**, **B**, Body weight was measured from mice bearing B16F10 mouse melanoma cells treated with control (vehicle) or 20 mg/kg lomitapide combined with either control isotype or anti–PD-1. Statistically significant differences was not calculated using an unpaired two-tailed Student’s *t* test (**A**). Representative H&E images of tissues (liver, kidney, and lung) collected from mice. Scale bar: 100 μm. (**B**)


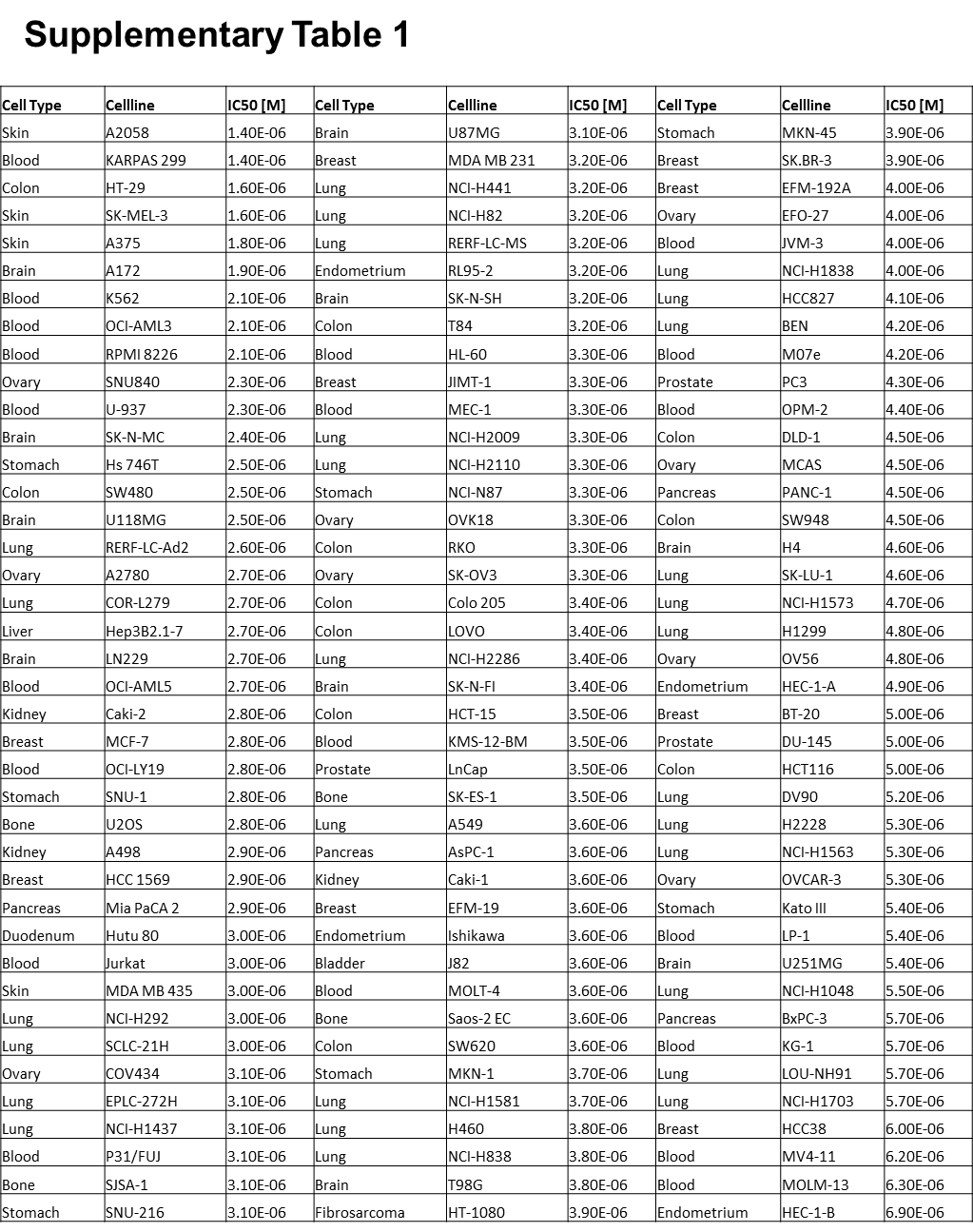


**Supplementary Table 1**

IC50 of 120 cancer cell lines treated 50 μM lomitapide for 24 h.


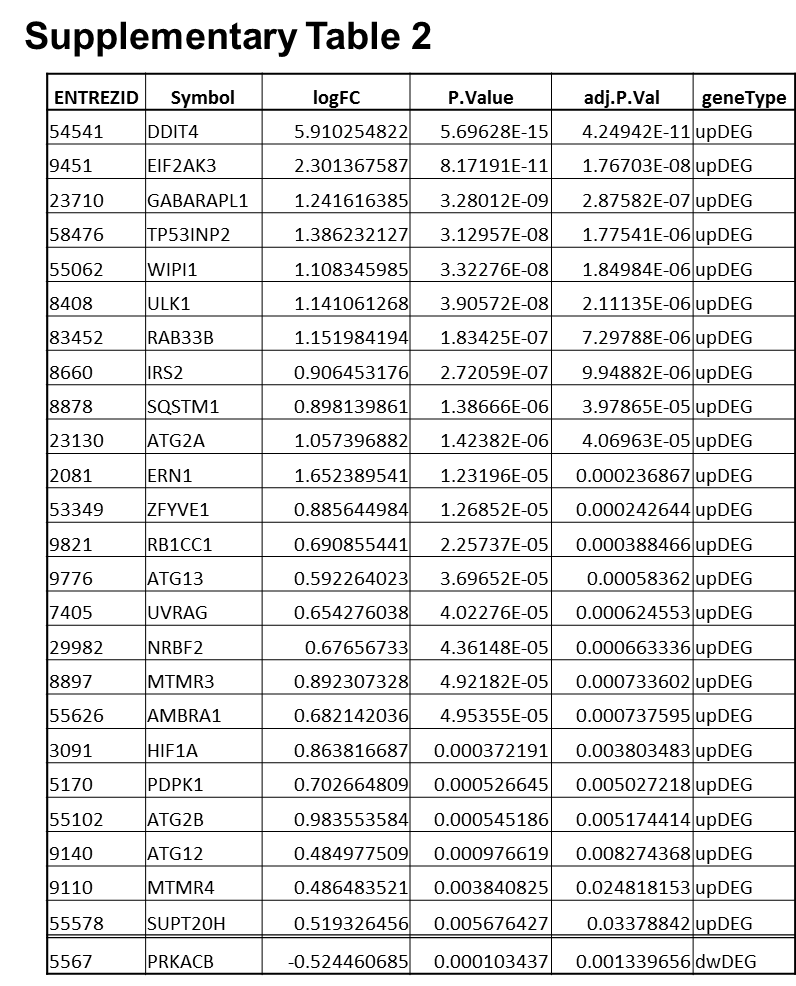


**Supplementary Table 2**

List of autophagy-controlling DEGs changed in lomitapide-treated HCT116 cells.
