# Supplementary material for: Lomitapide, a cholesterol-lowering drug, is an anticancer agent that induces autophagic cell death via inhibiting mTOR
Source: Cell Death Dis. 2022 Jul 12;13(7):603. doi: 10.1038/s41419-022-05039-6 (PMC9279289; doi:10.1038/s41419-022-05039-6)

Fig 1F


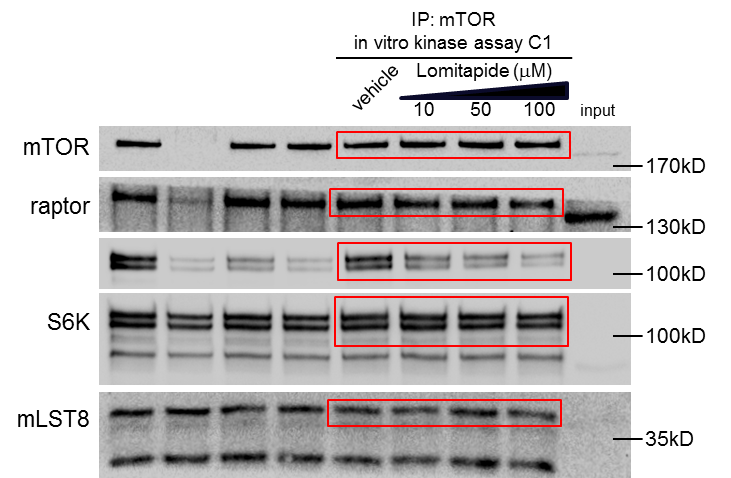


Fig 1G


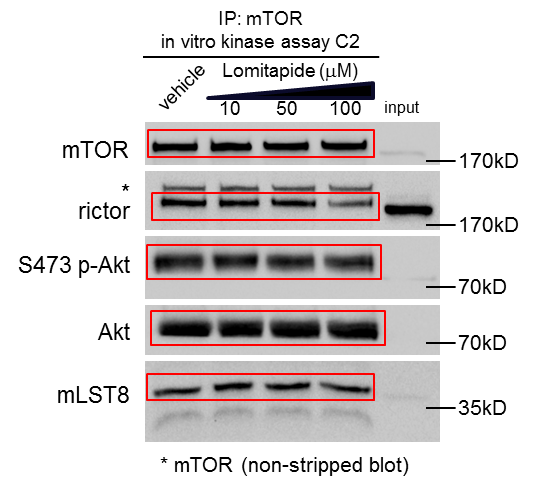


Fig 1H


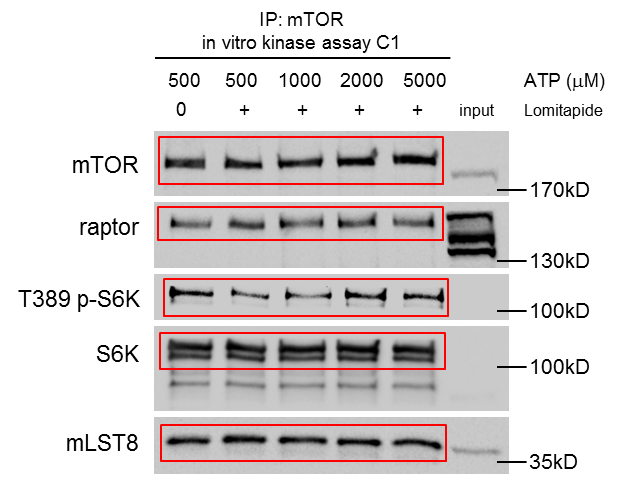


Fig 2C


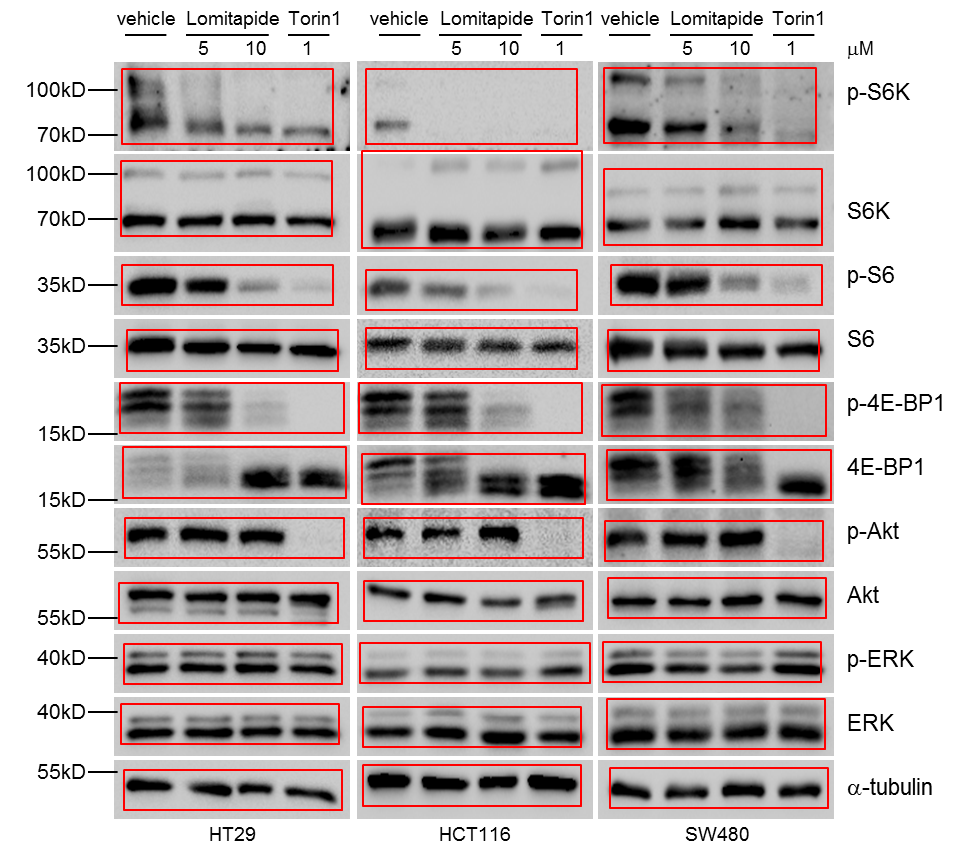


Fig 2D


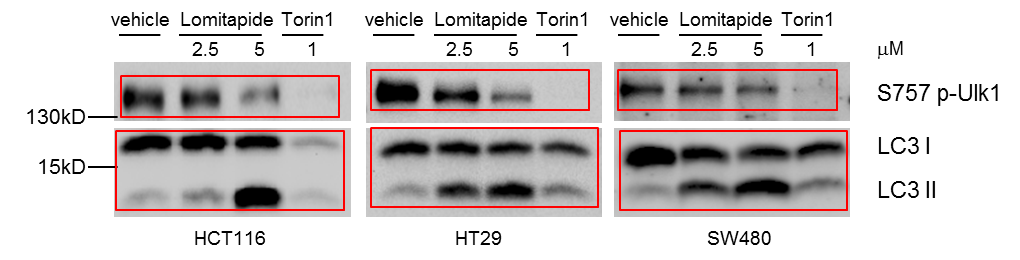


Fig 3E


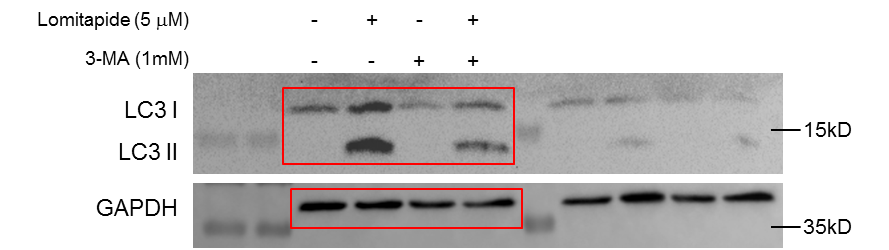


Fig 3F


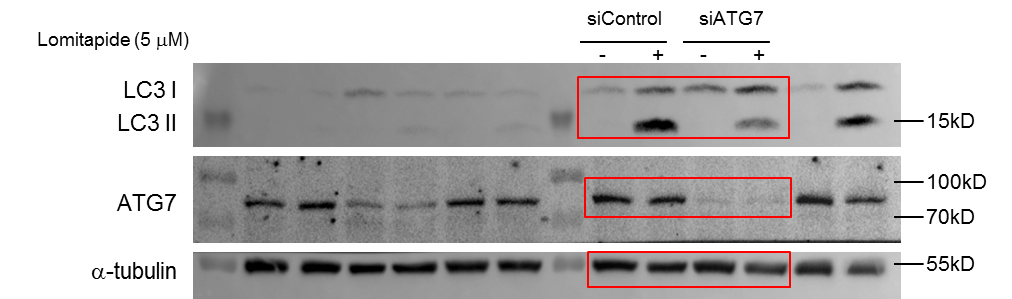


Fig 5D


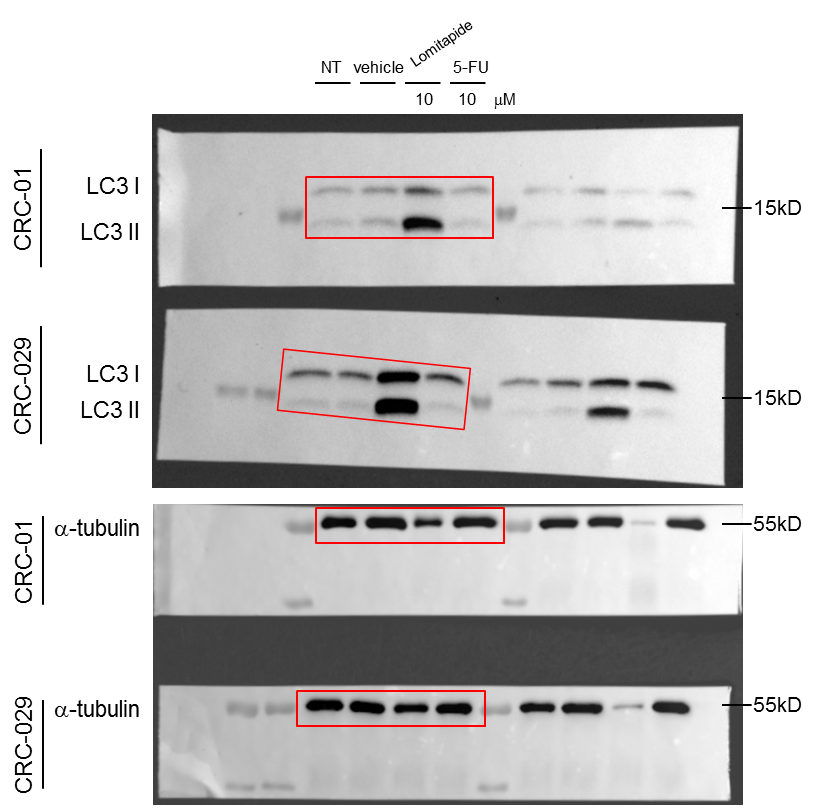


Supplementary FigS2


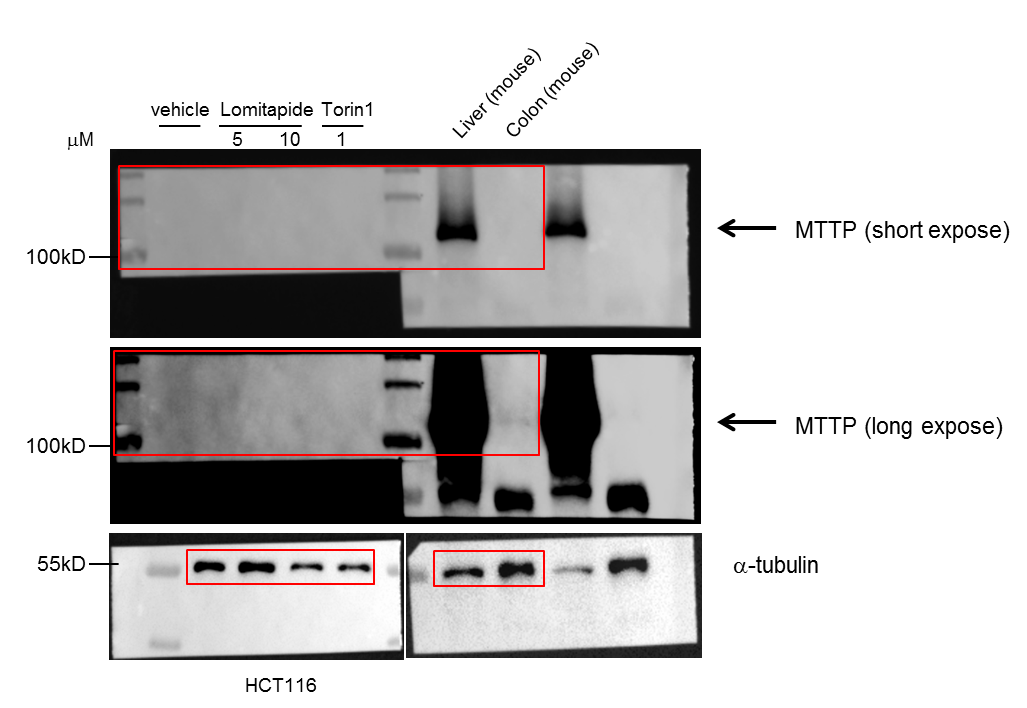


Supplementary FigS3


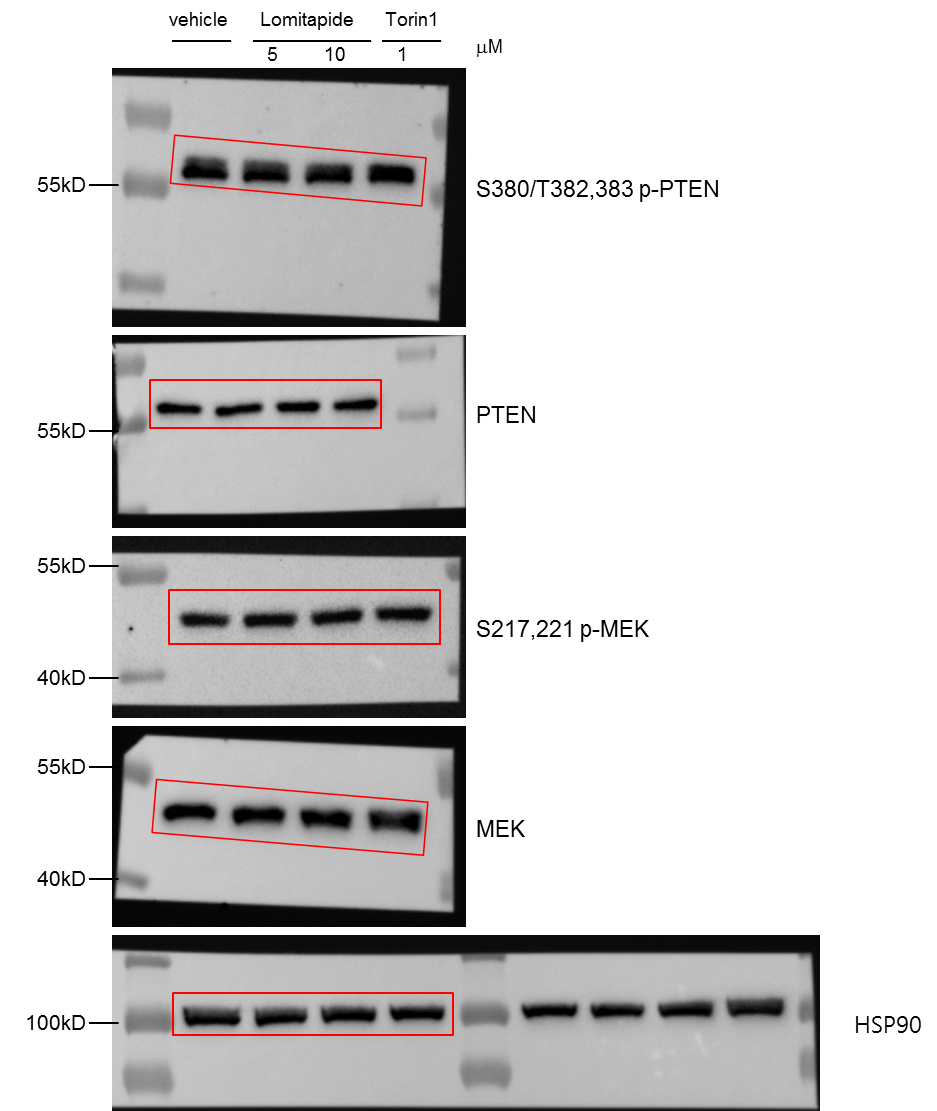


Supplementary FigS4A


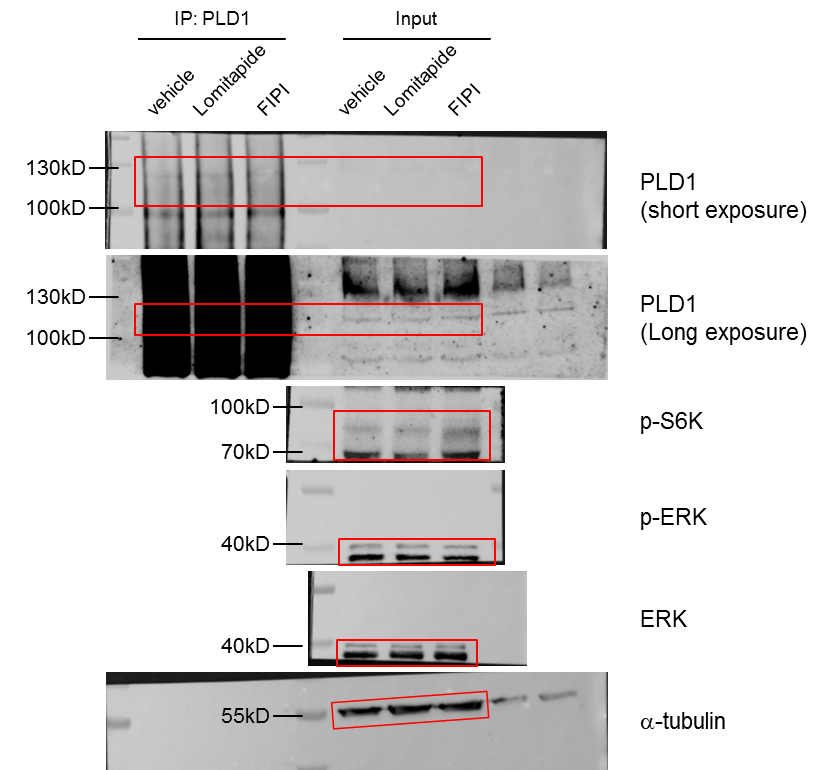


Supplementary FigS4C


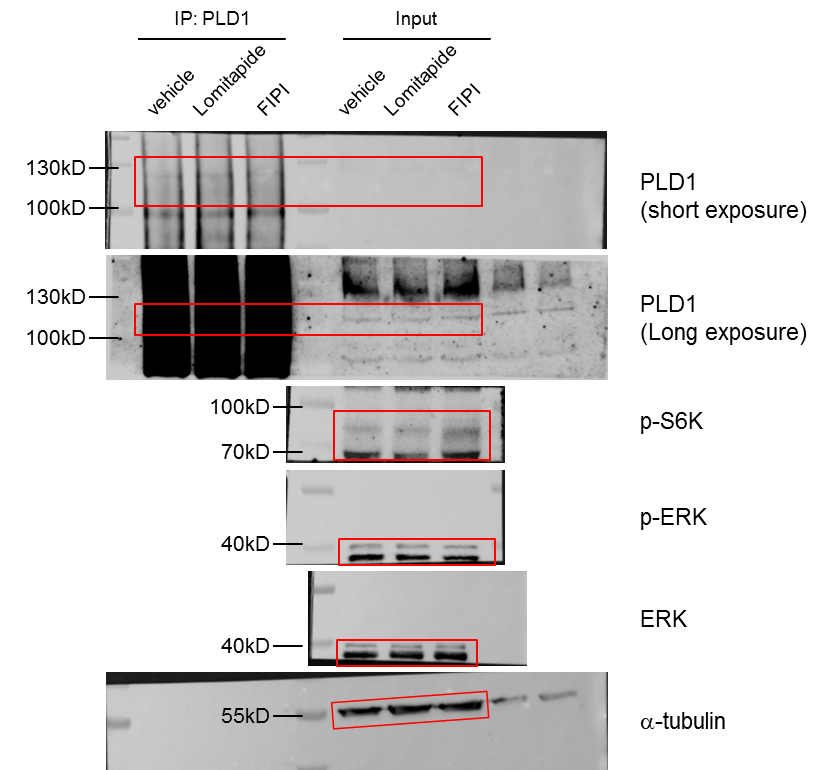


Supplementary FigS5_up_left


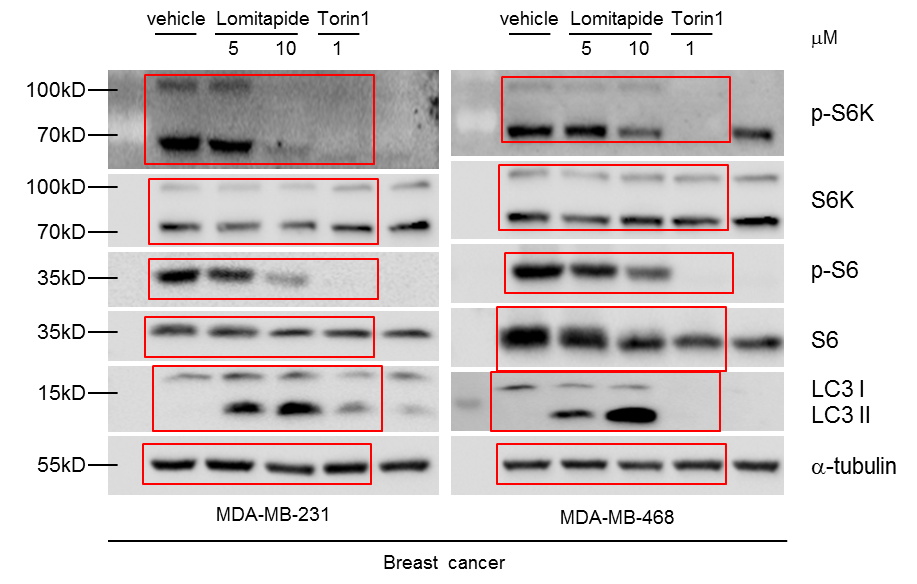


Supplementary FigS5_up_right


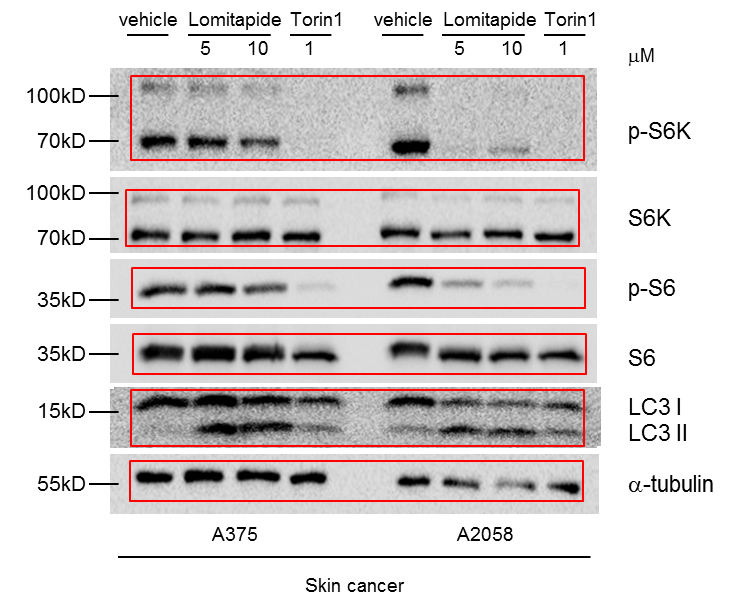


Supplementary FigS5_bottom


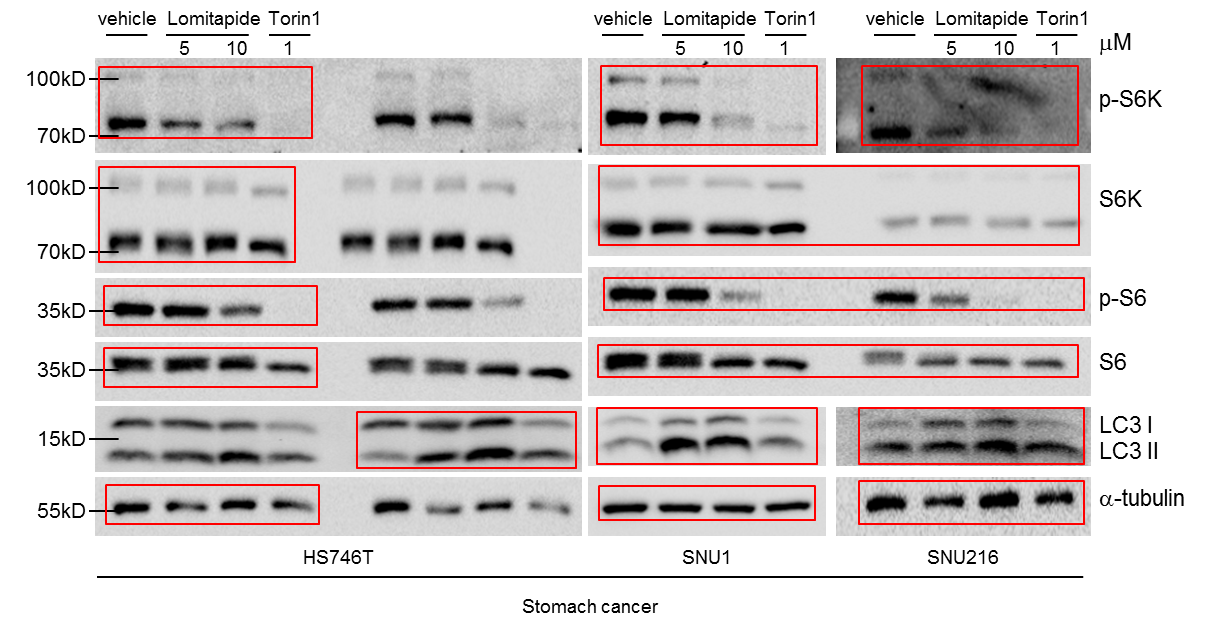


Supplementary FigS6D


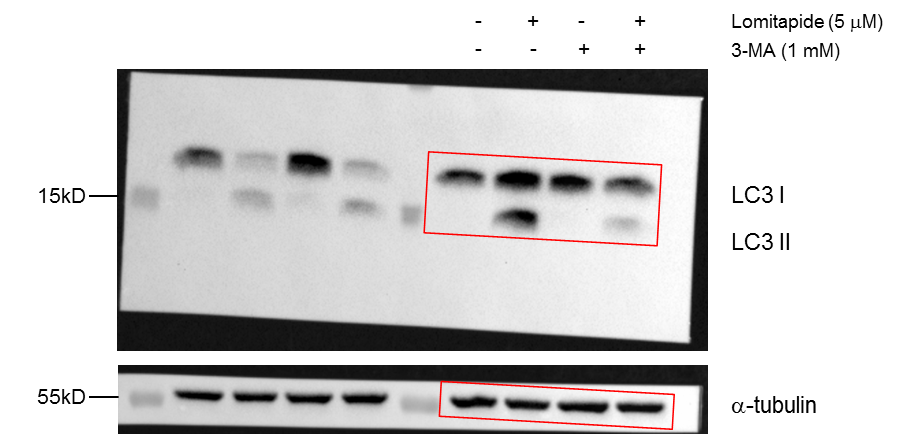


Supplementary FigS6E


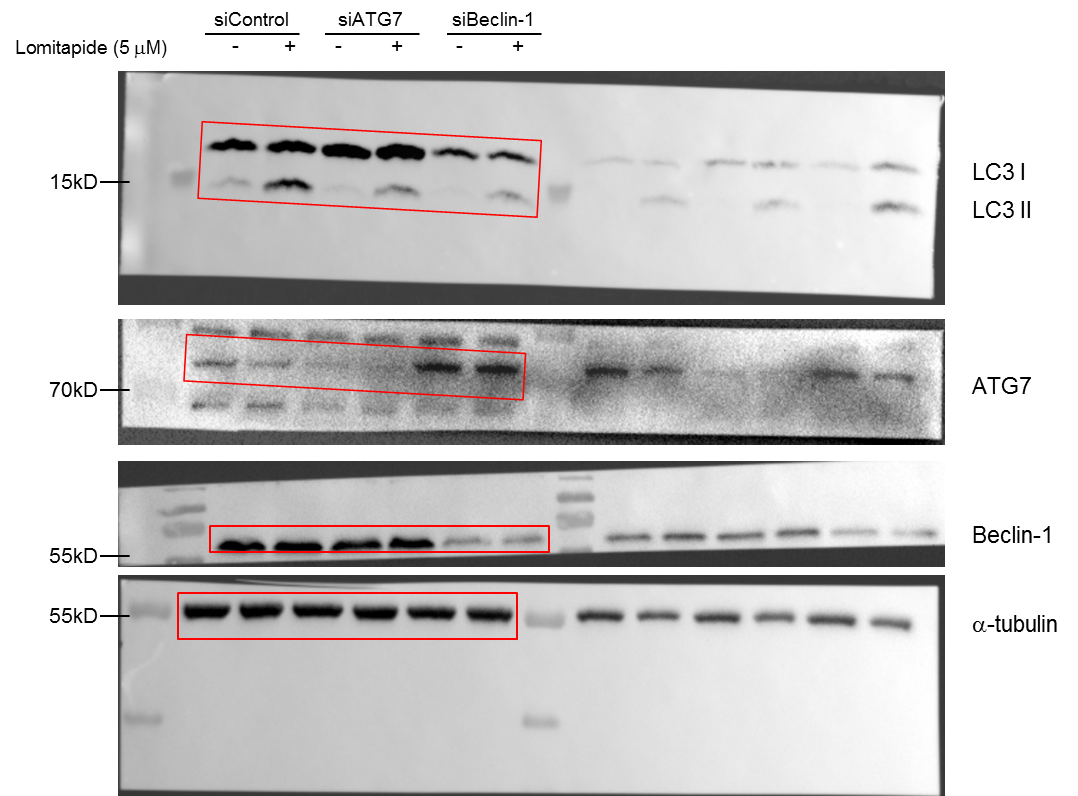


Supplementary FigS7


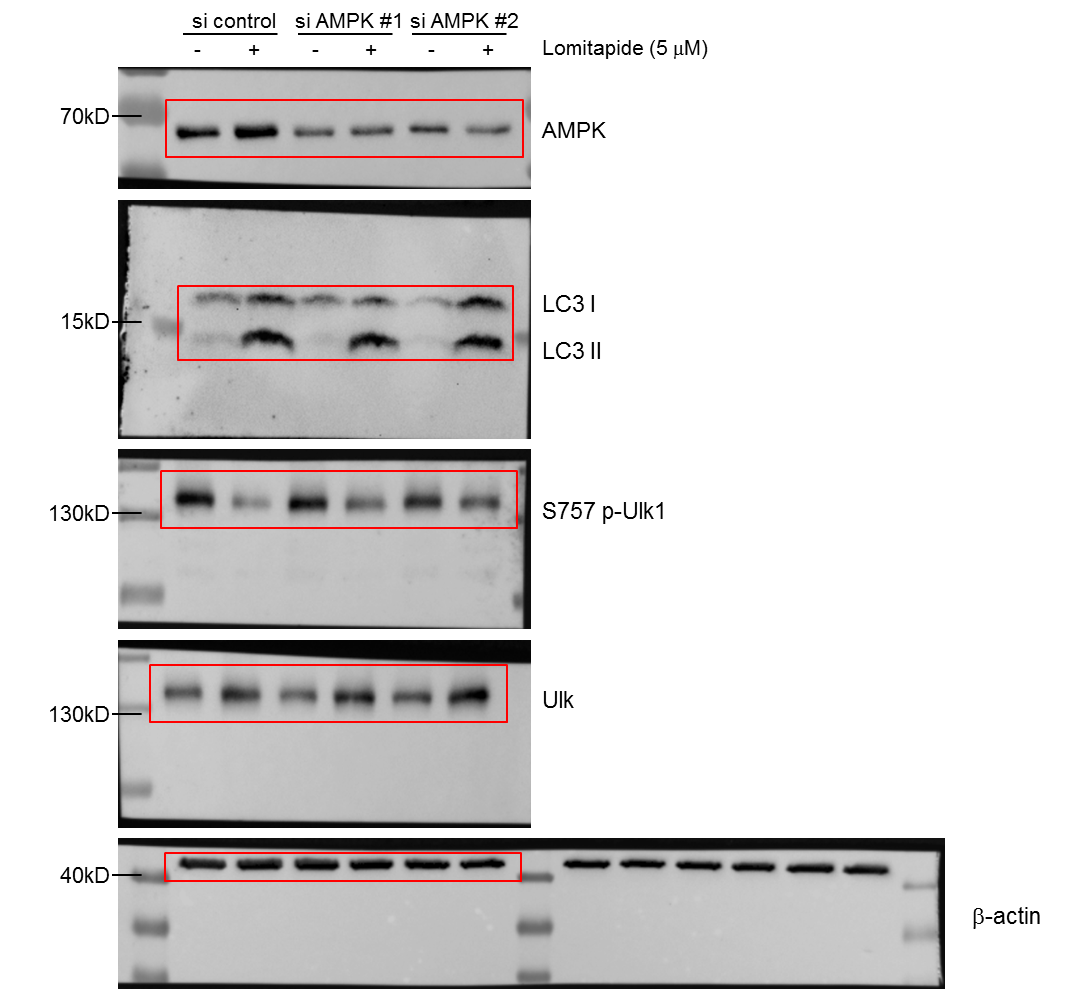


Supplementary FigS9A


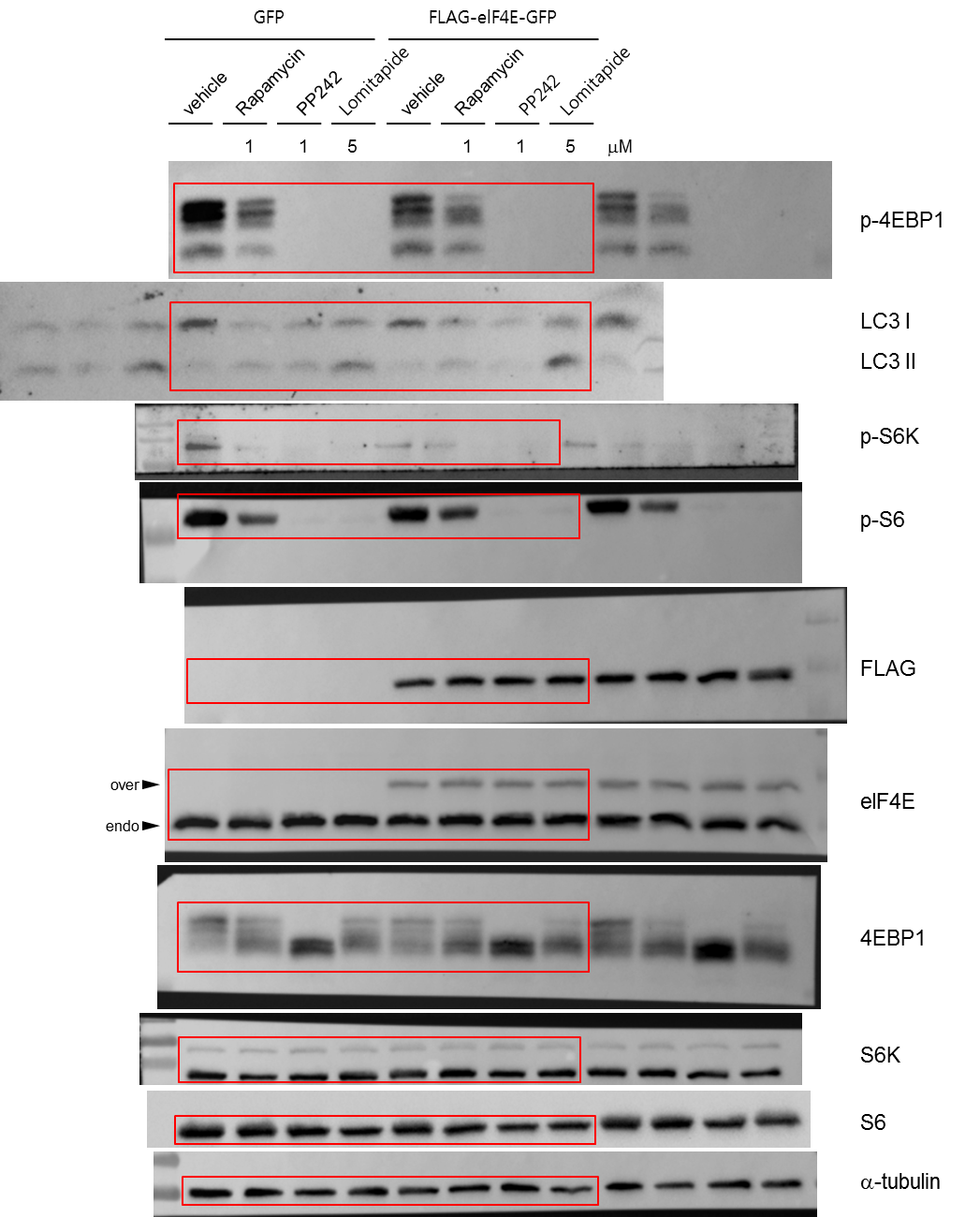

Supplement: Supplementary file 2 — Original Data File [file 41419_2022_5039_MOESM2_ESM.docx]
